# Metagenomic study of the microbiome and key geochemical potentials associated with architectural heritage sites: a case study of the Song Dynasty city wall in Shou County, China

**DOI:** 10.3389/fmicb.2024.1453430

**Published:** 2024-10-25

**Authors:** Mingyi Zhao, Yanyu Li, Huanhuan Chen, Yile Chen, Liang Zheng, Yue Wu, Kang Wang, Zhao Pan, Tao Yu, Tao Wang

**Affiliations:** ^1^Faculty of Humanities and Arts, Macau University of Science and Technology, Taipa, Macao SAR, China; ^2^Shanghai Biogenuinetech Co., Ltd., Shanghai, China; ^3^College of Life Sciences, Qingdao University, Qingdao, China; ^4^School of Art and Design, Shandong Jiaotong University, Changqing University Science and Technology Park, Jinan, China; ^5^Institutes for Translational Medicine, Qingdao University, Qingdao, China; ^6^The Affiliated Hospital of Qingdao University, Qingdao, China

**Keywords:** architectural heritage, ancient wall of Shou County, metagenome, biocorrosion, biogeochemical cycling

## Abstract

Historical cultural heritage sites are valuable for all of mankind, as they reflect the material and spiritual wealth of by nations, countries, or specific groups during the development of human civilization. The types and functions of microorganisms that form biofilms on the surfaces of architectural heritage sites influence measures to preserve and protect these sites. These microorganisms contribute to the biocorrosion of architectural heritage structures through the cycling of chemical elements. The ancient city wall of Shou County is a famous architectural and cultural heritage site from China’s Song Dynasty, and the protection and study of this site have substantial historical and cultural significance. In this study, we used metagenomics to study the microbial diversity and taxonomic composition of the Song Dynasty city wall in Shou County, a tangible example of Chinese cultural heritage. The study covered three main topics: (1) examining the distribution of bacteria in the biofilm on the surfaces of the Song Dynasty city wall in Shou County; (2) predicting the influence of bacteria involved in the C, N, and S cycles on the corrosion of the city wall via functional gene analysis; and (3) discussing cultural heritage site protection measures for biocorrosion-related bacteria to investigate the impact of biocorrosion on the Song Dynasty city wall in Shou County, a tangible example of Chinese cultural heritage. The study revealed that (1) the biofilm bacteria mainly belonged to Proteobacteria, Actinobacteria, Cyanobacteria, Bacteroidetes, and Firmicutes, which accounted for more than 70% of the total bacteria in the biofilms. The proportion of fungi in the microbial community of the well-preserved city wall was greater than that in the damaged city wall. The proportion of archaea was low—less than 1%. (2) According to the Shannon diversity index, the well-preserved portion of the ancient city wall had the highest diversity of bacteria, fungi, and archaea, and bacterial diversity on the good city wall was greater than that on the corroded city wall. (3) Bray–Curtis distances revealed that the genomes of the two good city walls were similar and that the genomes of the corroded city wall portions were similar. Researchers also detected human intestine-related bacteria in four locations on the city walls, with the proportion of these bacteria in the microbial community being greater on good city walls than on bad city walls. (4) KEGG functional analysis revealed that the energy metabolism and inorganic ion transport activities of the bacterial community on the corroded city wall were greater than those of the good city wall. (5) In the carbon cycle, the absence of active glycolysis, the ED pathway, and the TCA cycle played significant roles in the collapse of the east city wall. (6) The nitrogen cycling processes involved ammonia oxidation and nitrite reduction to nitrate. (7) In the sulfur cycle, researchers discovered a crucial differential functional gene, SoxY, which facilitates the conversion of thiosulfate to sulfate. This study suggests that, in the future, biological approaches can be used to help cultural heritage site protectors achieve targeted and precise protection of cultural relics through the use of microbial growth inhibition technology. The results of this study serve as a guide for the protection of cultural heritage sites in other parts of China and provide a useful supplement to research on the protection of world cultural heritage or architectural heritage sites.

## 1 Introduction

### 1.1 The role of microorganisms at architectural heritage sites cannot be ignored

Cultural heritage sites left behind by previous civilizations are valuable assets for all of humanity, as they reflect the material and spiritual wealth of nations, countries, or specific groups during their development. In recent years, the protection of cultural heritage sites has received extensive attention in various fields worldwide and has sparked profound discussions in academia. China, as an ancient and culturally rich Eastern country, has abundant cultural heritage sites. City walls are the most prominent material symbols of ancient Chinese cities and constitute one of the core elements of ancient Chinese urban civilization. The ancient city wall in Shou County, the object of this study, is one of fewer than 20 relatively intact ancient city walls in China. The oldest part of the wall was built in the Song Dynasty, and most of the wall was rebuilt or repaired during the Ming and Qing Dynasties. In 2006, the city wall of Shou County, together with seven other city walls in China, was successfully included in the joint application project “Chinese Ming and Qing City Walls” and listed on the “China World Heritage Tentative List” (He and Chen, 2021).

However, over the course of its long history, Shou County’s city wall has experienced varying degrees of damage, placing some of the ancient walls in danger. On the one hand, changes in the climate and environment lead to weathering and damage to ancient city walls and other similar architectural relics (Basu et al., 2020; Brimblecombe et al., 2010). On the other hand, the increase in human activity and resulting changes further complicate the survival and preservation of these ancient structures (McNeill, 2009). Microorganisms, which are closely related to human life and influence each other in ancient buildings, represent a research direction worthy of attention and exploration (Stanaszek-Tomal, 2020; Liu et al., 2020; National Academies of Sciences, 2017). Contemporary microorganisms attached to building surfaces not only degrade the beauty of architectural relics but also may corrode and damage building materials, accelerating the demise of ancient buildings. Because of this, biologists have performed relevant experiments to test and monitor the microbial populations in old building remnants, to learn about their traits, and to come to reasonable conclusions about what could be done to stop deterioration and improve preservation in the future on the basis of the results of the experiments. Their findings present new scientific approaches and ideas for the diagnosis and protection of architectural heritage sites. At the same time, researchers recognize that currently, exploration and research in the field of microorganisms in architectural heritage sites are lacking in both quantity and depth and is still in its early stages. Researchers hope that this exploration will provide meaningful help for the future maintenance and restoration of architectural cultural heritage sites.

### 1.2 Ecological characteristics of microbial communities in architectural heritage sites

Microorganisms play a variety of important roles in nature and in all human activities (Gadd, 2010). Microorganisms are also closely related to biocorrosion in cultural heritage sites, including ancient buildings, and this has drawn the attention of the academic community for a long time (Sanmartín et al., 2018). Current research reveals that certain microorganisms can lead to specific types of corrosion in building materials (Stanaszek-Tomal, 2024; Nowicka-Krawczyk et al., 2022), prompting the development of strategies for mitigating these effects (Romani et al., 2022; Sánchez et al., 2023). Microorganisms also have an impact on ancient buildings. Metagenomic research can be used to analyze the consequences and causes of microbial processes on architectural heritage sites (Skipper et al., 2022; Negi and Sarethy, 2019). Notably, existing studies have suggested that environmental changes such as air flow and increased exhaust gas cause changes in microbial abundance and their mechanisms of action on buildings (Gaylarde et al., 2018; Boniek et al., 2021). Although the main building material of the ancient city wall in Shou County is relatively stable brick, the wall has still undergone corrosion. Therefore, it is necessary to use biotechnological methods to study the ancient city wall in Shou County, analyze the level of corrosion, and formulate maintenance measures. Researchers have demonstrated that microorganisms can be used to stabilize building materials and repair cultural heritage structures under specific conditions (Pyzik et al., 2021; He et al., 2022). However, the number of such studies, whether involving the investigation of architectural heritage sites through biological means or the restoration and protection of buildings with the help of biotechnology, is still relatively small. We hope that the ideas, methods, and viewpoints of this research can serve as references for the protection and study of similar architectural heritage sites and contribute to the protection of world architectural heritages.

On the other hand, the biodeterioration of ancient buildings is closely related to microorganisms, and the ancient city wall of Shou County, which is primarily made of relatively stable bricks, is no exception. Many bricks from cultural heritage sites, such as brick steles and ancient brick structures, are believed to have been subject to physical and chemical damage for long periods (Miller et al., 2012; Sterflinger and Piñar, 2013). In addition, biological influences have been considered in recent decades because of the bioreceptivity of building materials (Miller et al., 2012). Bacteria, archaea, and fungi play important roles in the biodeterioration of structures (Gadd, 2017; Liu et al., 2022; McNamara and Mitchell, 2005) via bioweathering (Gadd, 2007), biocorrosion (Skipper et al., 2022), and biodegradation (Scheerer et al., 2009), altering the structural composition of building materials (Polynov, 1945). Biological influences are usually linked to microbial colonization and biofilm formation (Jain et al., 2007; Prakash et al., 2003; Liu et al., 2020), and the mechanisms of biofilm formation during microbial colonization are key to revealing the drivers of biodeterioration (Sanmartín et al., 2021).

The physical mechanisms of bioweathering include various processes (Allsopp et al., 2004; Zhang et al., 2019; Gu and Katayama, 2021), which include penetration by filamentous microorganisms (such as certain actinobacteria, cyanobacteria, algae, and fungi), as well as nonfilamentous cyanobacteria along weak points. Additionally, direct tunneling or boring, particularly in weakened or porous substrata, contributes to bioweathering (Bin et al., 2008). Furthermore, the mineral lattice is weakened through wetting and drying cycles, followed by expansion and contraction, whereas the production of secondary minerals is facilitated by the reaction of anions from microbially secreted acids with cations from the bricks (Fan et al., 2019; Hughes and Bargh, 1982). The biochemical weathering of rock can be initiated through the microbial secretion of organic and inorganic acids, siderophores, and other metabolites (Turick and Berry, 2016). Sulfur-and sulfidic-oxidizing bacteria, such as Acidithiobacillus spp., are widely acknowledged for their ability to cause deterioration of sulfidic substrates, concrete, and bricks (Cwalina, 2014). Similarly, sulfate-reducing bacteria (SRB), such as Acidithiobacilli, play a significant role in the biodeterioration of concrete (Turick and Berry, 2016; Ul-Abdin et al., 2022). Additionally, many bacteria, especially anaerobes, can utilize alternative electron acceptors for respiration, including NO_3_^−^, SO_4_^2−^, Fe(III), and Mn(IV) (Kim and Gadd, 2008; Sokatch, 2014), leading to the reduction (or oxidation) of such components in minerals, ultimately causing instability and dissolution (Uroz et al., 2009). Thus, the microbial community composition, diversity and mechanisms related to biodeterioration in biofilms on brick cultural heritage sites are increasingly important for the structural preservation of historical sites (Price and Doehne, 2011; Tyagi et al., 2021).

Despite the widespread use of 16S amplicon sequences to profile the microbiome via small rRNA gene units in microbial community ecology research, the microbiomes of cultural heritage sites have not been extensively investigated via metagenomics (Vaudour et al., 2015; Sáiz-Jiménez, 2015). Specifically, the library obtained from amplicon sequencing analysis is not uniform in terms of the PCR efficiency of universal primers used for the 16S rRNA target region and is easily influenced by the sequence of the amplification target region, leading to underamplification, thereby causing distortion in community composition analysis (Wu et al., 2023). The taxonomic and functional compositions of cultural heritage biofilms and the mechanisms of biofilm-driven biodeterioration at the gene level are poorly understood.

### 1.3 Problem statement and objectives

**Figure 1:**
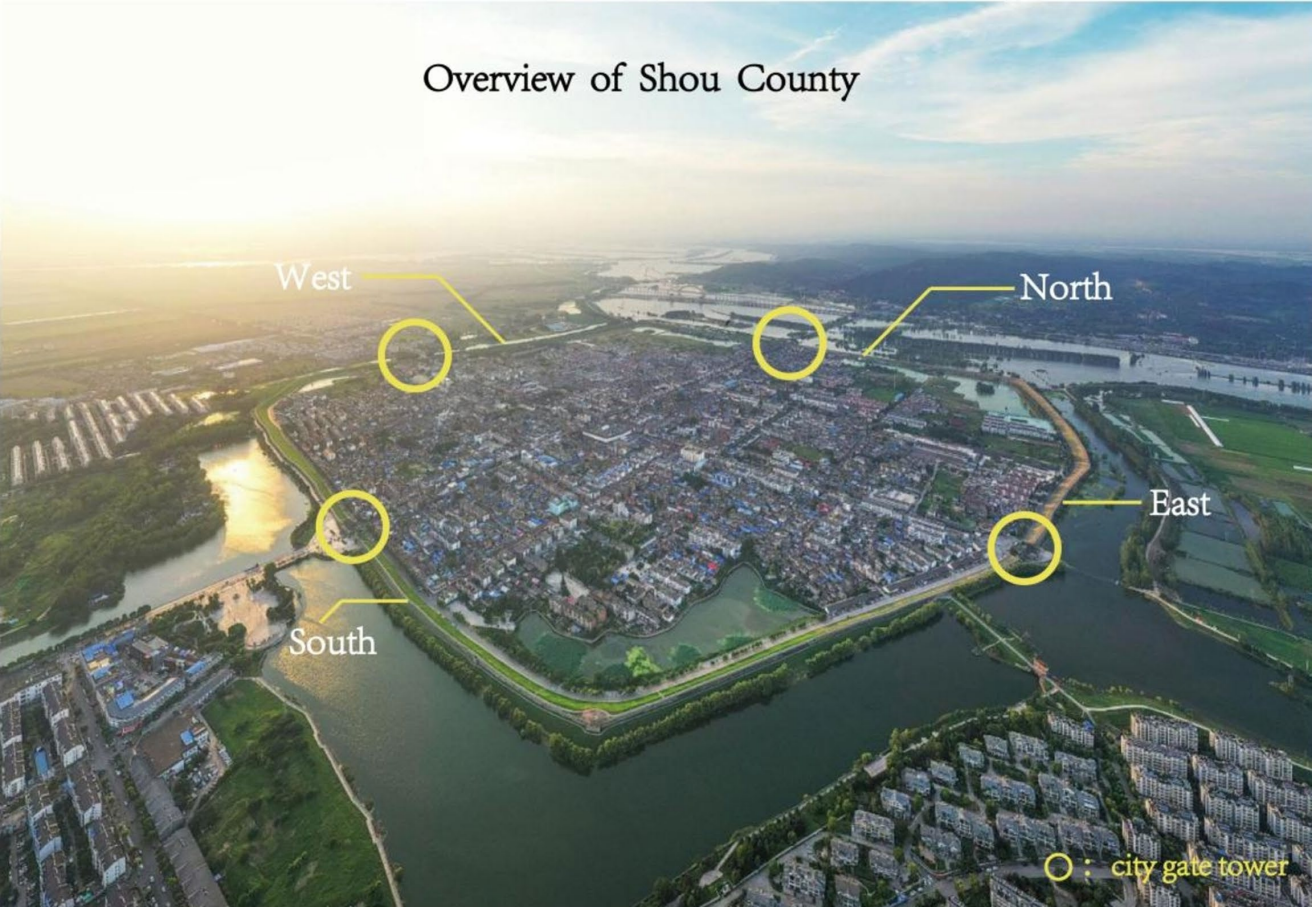
Overview of Shou County.

**Figure 2:**
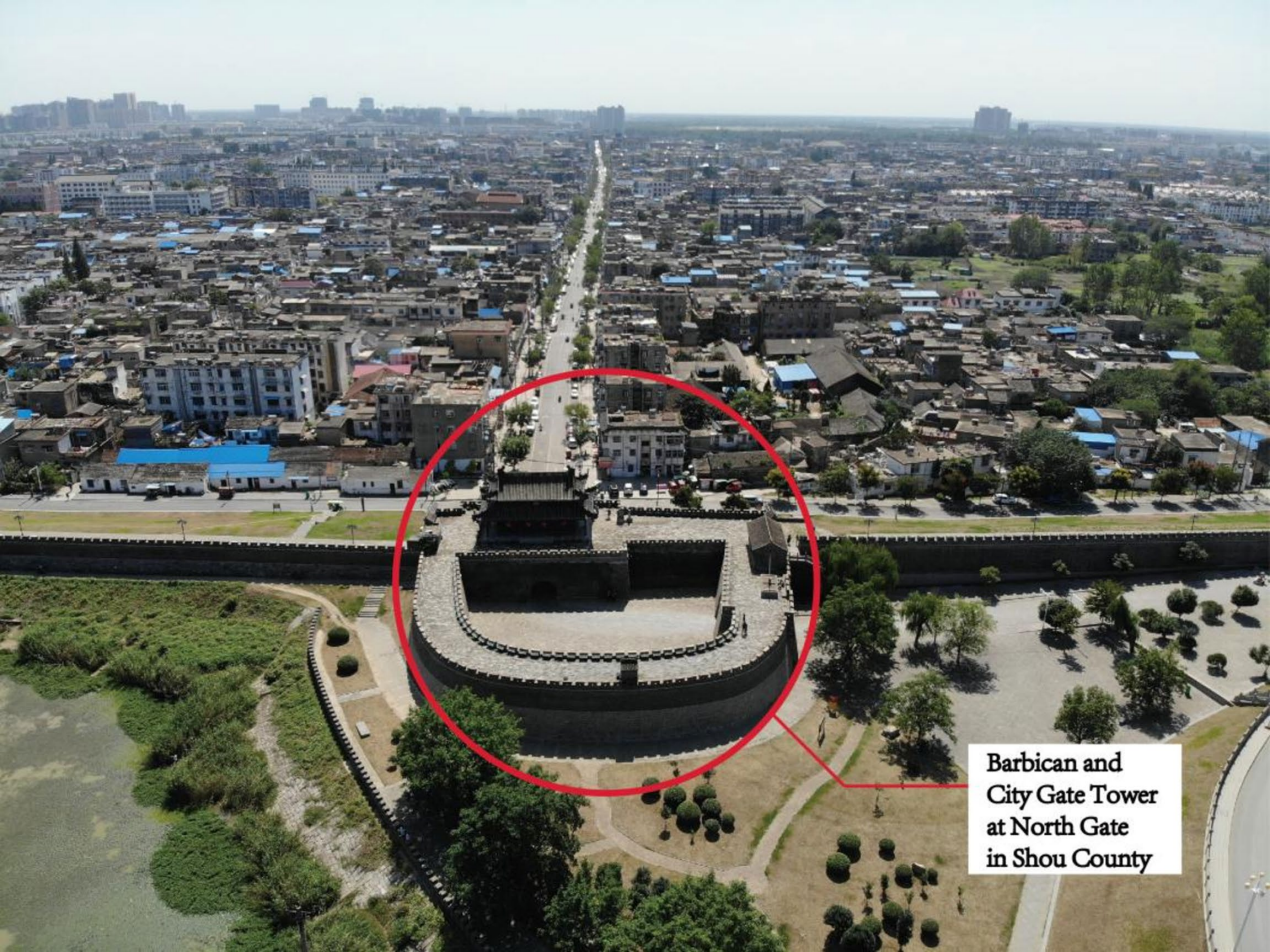
The barbican and city gate towers at the North Gate in Shou County.

Thousands of years after they were built, the outer walls of the ancient city in Shou County remain basically intact (Figure 1), and people still live in the city. Towers are located in the middle of city walls in four directions: east, west, south, and north. The towers still retain the common barbican architectural form developed in ancient China to defend against enemies (Figure 2). Today, some architectural ruins from the Song Dynasty are still visible on the east city wall and tower. Most of the walls and towers of the northern city wall were built during the Ming Dynasty, they were repaired during the Qing Dynasty, and modern maintenance has kept them intact. In modern times, the west city wall and tower were completely reconstructed. The condition of the south city wall and tower are similar to that of the west city wall. Owing to their advanced age and the influence of human activities, the ancient city walls of Shou County have contributions from different historical periods. Some ancient microorganisms and modern microorganisms attached to the surface of the bricks of the ancient city wall have affected the beauty of the wall and caused corrosion and damage to the bricks, and some of the ancient walls are in danger of collapse.

Therefore, in this study, we investigated the following topics. (1) First, we collected samples of bricks from the severely corroded east wall in Shou County near the residential area, the better preserved inner part of the barbican (the gates of Chinese city walls were often defended by an “archery tower” that was located in front of the main gatehouse, with the two towers connected by walls extending out from the main fortification), the severely corroded inner part of the north wall, and the newly rebuilt west wall near the residential area. These samples were then subjected to metagenomic sequencing. (2) On the basis of the metagenomic sequencing results of the brick samples from four different locations, we obtained the specific composition of bacteria in the biofilms on the surfaces of the bricks of the ancient city in Shou County, which has existed for thousands of years. (3) Using bacterial community comparisons and functional annotation, we analyzed the distribution of microorganisms, functional genes, and elemental cycling pathways in the biofilm on the brick surface. (4) Through functional gene analysis, we predicted the influence of bacteria involved in the carbon, nitrogen, and sulfur cycles on the corrosion process of the wall and identified key elemental cycles and related microorganisms that affect the corrosion process of the wall bricks. (5) Finally, on the basis of specific microbiological findings, we discuss protection measures for the architectural heritage structures and explore the impact of biological corrosion of the ancient city wall in Shou County. Our findings reveal the diversity and potential metabolic activities of microbial communities in the unique environment of the ancient city wall of Shou County, China, providing valuable insights into cultural heritage site maintenance and protection.

## 2 Materials and methods

### 2.1 Site description and sampling

#### 2.1.1 Research objects: Song Dynasty City walls in Shou County

**Figure 3:**
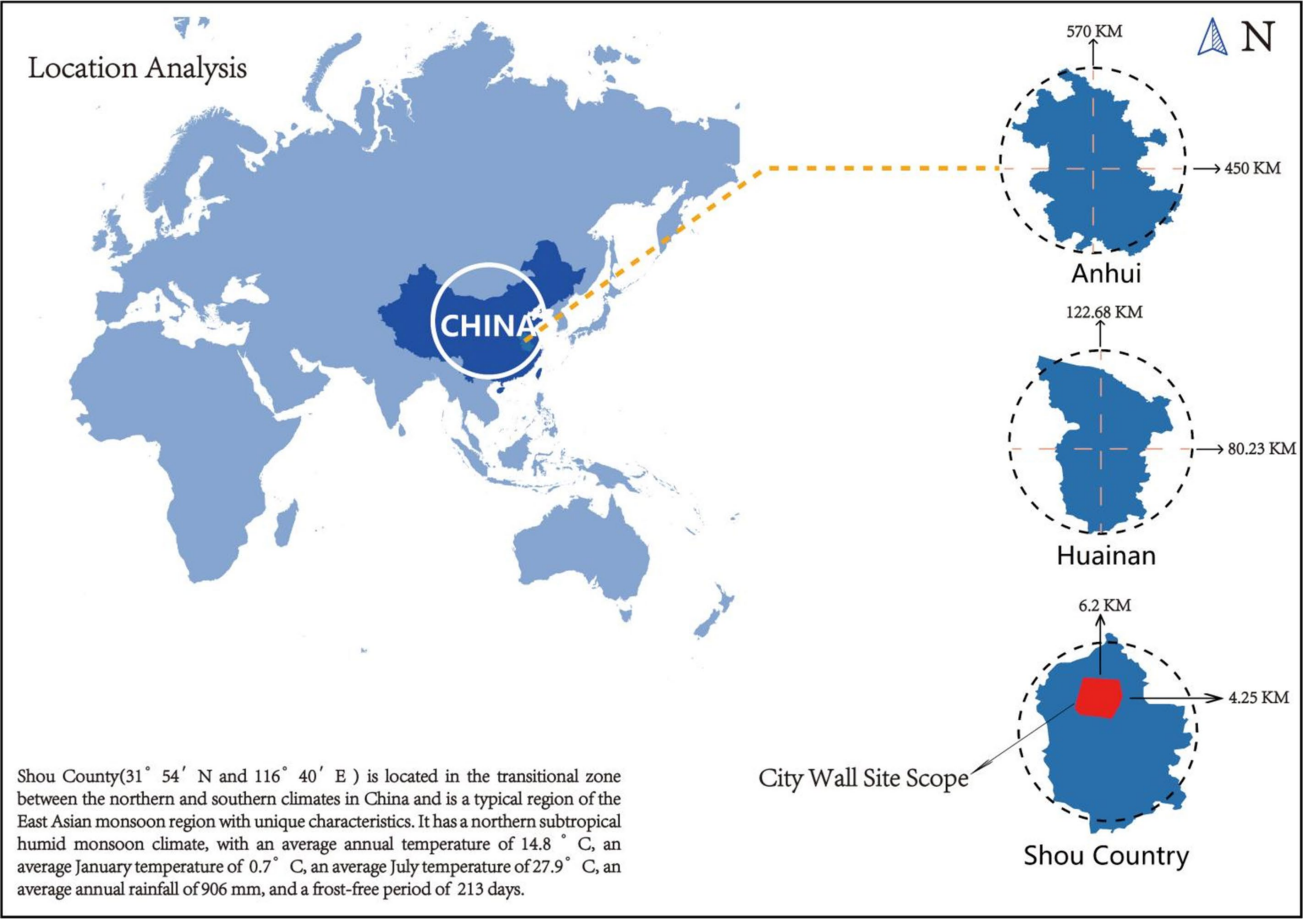
Location analysis of Shou County (N 31°54′, E 116°40′).

Biofilm samples from the surface of the ancient city walls were collected in Shou County in Anhui Province, East China. The walls of the ancient city in Shou County, one of seven well-preserved ancient city walls in China, are located in an ancient Song city with a checkerboard layout on the southern side of the Huai River in Shan County (N 31°54′, E 116°40′) (Figure 3). The ancient city originated from the capital of the Chu State (B.C. 241) and suffered continuous damage due to conflicts such as the Battle of Feishui and floods, which do not occur in the present day. The South Gate and West Gate were damaged and restored around the year 2000, whereas the North Gate and East Gate were rebuilt during the Northern Song Xining to Southern Song Jiading period (A.D.1068–1,224), which spanned more than 900 years.

#### 2.1.2 Climatic conditions and characteristics of the City Wall structure

Shou County is located in the transitional zone between the northern and southern climates in China and is a typical area representing the East Asian monsoon region with unique characteristics. It has a northern subtropical humid monsoon climate, with an average annual temperature of 14.8°C, an average January temperature of 0.7°C, an average July temperature of 27.9°C, an average annual rainfall of 906 mm, and a frost-free period of 213 days (please refer to Appendix A at the end of this article for a detailed Ladybug analysis climate map). Owing to climatic differences, in ancient Chinese civilizations, city walls were built with rammed earth in the north and masonry in the south. Shou County, located at the intersection of northern and southern China, has a city wall that is half made of rammed earth and half made of masonry, making it a “slope battle city.” Masonry forms the outer wall of the city wall, whereas rammed earth forms the inner wall, creating a slope-like structure. This structure provides a transportation advantage to military defensive units, allowing for multiple troops in the city to fight at the same time. Moreover, prior to the Song Dynasty, the Huaihe River’s water flowed into the sea.

However, during the fifth year of Shaoxi in the Southern Song Dynasty (A.D. 1,194), the Yellow River surged southward, engulfing the Huai River. This led to silting of the Huai River estuary, frequent floods, and disturbances in Shou County. The wall of the “slope battle city” was fortified with a thick layer of rammed earth, and for defense, a brick wall layer filled with glutinous rice lime was employed, thereby enhancing the wall’s waterproofness and impermeability. In modern times, several large-scale renovations have been carried out on the Shou County city wall. There are four gates in the city: the East “Binyang” Gate, the South “Tongfei” Gate, the West “Dinghu” Gate, and the North “Jinghuai” Gate, all of which have barbicans. The East Gate’s gatehouse was reconstructed in the 1990s, and the oldest parts of the city wall in Shou County from the Song Dynasty consist of the city wall and the gate culvert. There are also traces of modern repairs and reconstructions in the barbican. Shou County’s main gate, the South Gate, has suffered severe damage. In approximately 2000, the existing South Gate in Shou County was rebuilt using old bricks and stones collected on-site and donated by the city’s residents, preserving its traditional charm during modern reconstruction. In recent years, the city wall and gatehouse of the West Gate were constructed utilizing new bricks and advanced technology. In 1976, the North Gate underwent modernization and reinforcement to maintain its blue brick wall in response to the need for flood control. Therefore, the wall properties of the four gates in Shou County are different.

#### 2.1.3 Sampling

**Figure 4:**
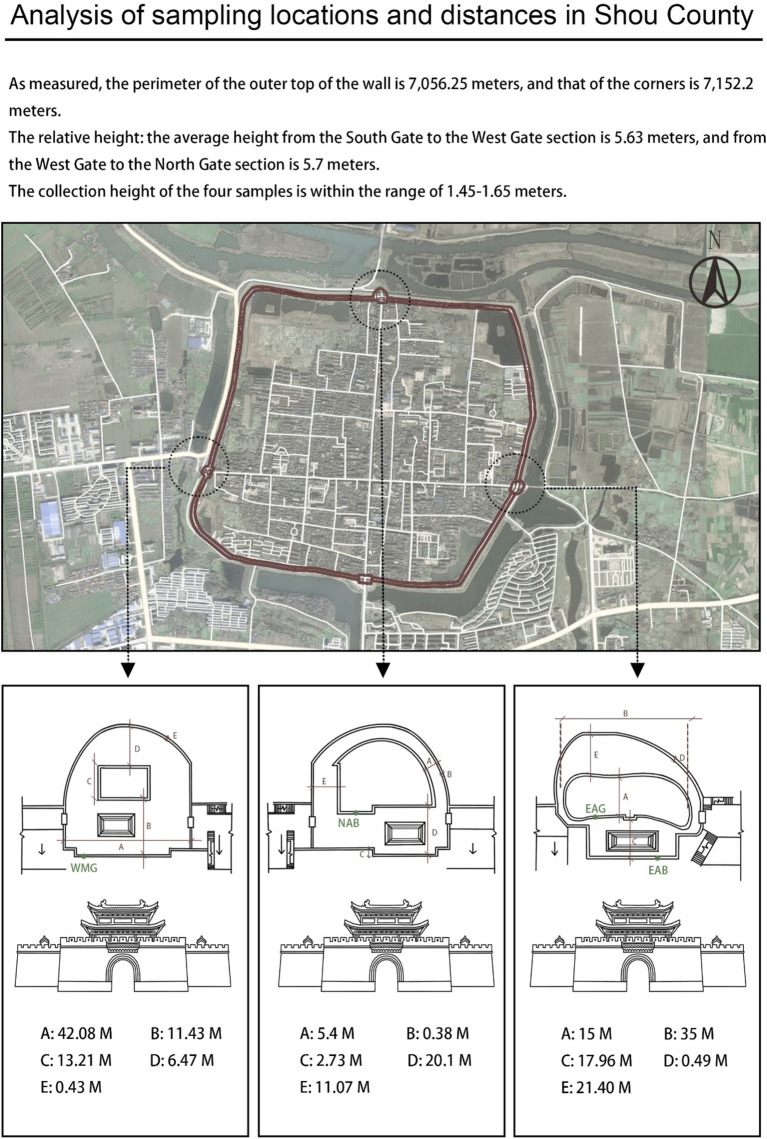
Analysis of sampling locations and distances in Shou County.

**Figure 5:**
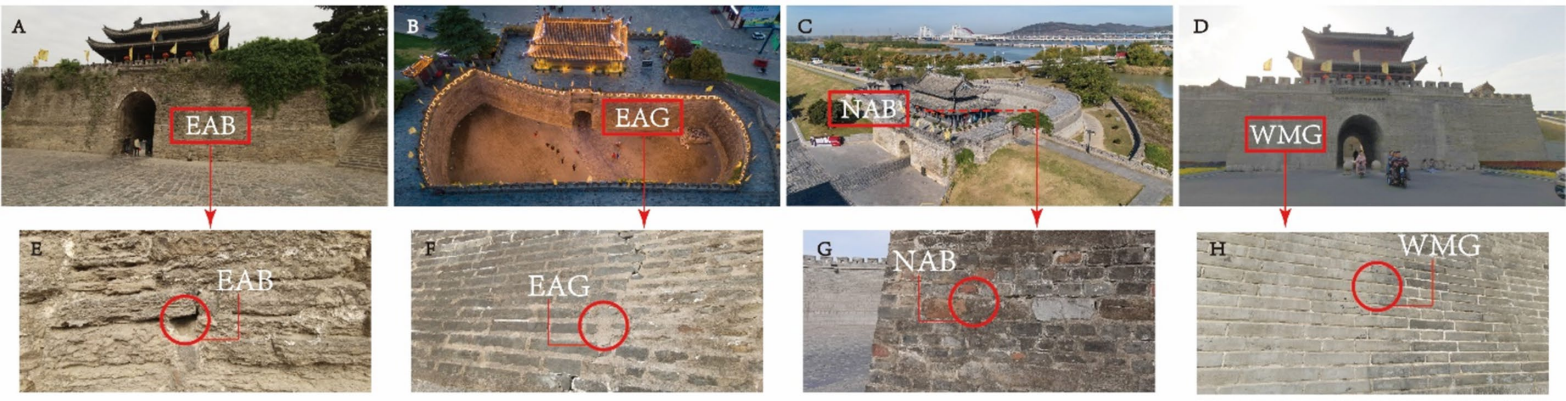
Overall appearance and view of the Shou County ancient city walls and the location and environmental characteristics of each study site. A-D, overall view of the ancient gates; E-F, sampling location at the East Gate of the ancient walls. G, Sampling location at the North Gate of the ancient walls. H, Sampling location at the West Gate of the modern walls.

On the basis of the characteristics of the sampling site, which is adjacent to a moat on the east side and has various transportation routes on the north side, researchers selected the ancient city walls on the east and north sides and the modern city walls on the west side for sampling. On April 26, 2023, at each sampling point, a sterile scalpel was used to carefully peel and collect samples from the surface of the city wall and from gaps between the bricks, and then a cotton swab was used to delicately collect the samples, which were placed in a sterile test tube and sealed. Sampling was carried out on the city walls of the East City Tower, North City Tower, and West City Tower in Shou County, and the sample collection height ranged from 1.45–1.65 m (that is, when the sampler was upright, the arm of the sampler was approximately at the height of a human’s head and shoulders). Figure 4 shows the specific locations and distances of the other samples. Given the recent reconstruction of the entire west wall and similar preservation measures, the intact wall bricks were randomly sampled and labeled West-Modern-Good (WMG). For east wall sampling, where the repair and damage of the ancient wall was complex, the most damaged brick joints of the ancient wall observable to the naked eye and the largest brick joints were sampled and labeled East-Ancient-Bad (EAB); these samples were selected to explore the reasons for the damage to the east ancient wall. At the same time, East-Ancient-Good (EAG) samples were taken from parts where the overall condition of the wall was good, but few repair marks were observable to the naked eye; these features clearly distinguished the EAG samples from the EAB samples. The north wall also survived many dynasties, and the repair status was complex; however, there were no large differences in the preservation forms, unlike in the east wall, so North-Ancient-Bad (NAB) samples were taken from the parts where the bricks of the wall were of different materials and colors and from cracks observable to the naked eye, and the wall surface and brick joints were sampled simultaneously during sampling (Figure 5). Permission for sample collection was granted by the Shou County Culture and History Office. Approximately 5 g of each sample material was collected, placed in a sterile 10 mL tube and stored in a cooler with a coolant for transportation. For omics analysis, the biofilm samples were transferred to Shanghai BioGenuineTech Co., Ltd. and stored at −80°C until further processing and analysis.

### 2.2 DNA extraction and DNBSEQ-T7 high-throughput sequencing

Total genomic DNA was extracted from each biofilm sample via a TIANamp Soil DNA Kit (TIANGEN BIOTECH, Beijing, China) according to the manufacturer’s instructions. The concentration and purity of the extracted DNA were determined via a Qubit4 instrument (Thermo Fisher Scientific, Illinois, United States). A protocol modified from one previously described (Rinke et al., 2016) was used for the construction of the paired-end sequencing library. Briefly, less than 1 ng (0.1 ng/ul-1 ng/ul) of extracted DNA from biofilm samples was fragmented by a transposase reaction mixture (5 μL of 2× TD buffer, 2.5 μL of transposase (BioGenuineTech, Shanghai, China), and 1.5 μL of nuclease-free water) for 10 min at 55°C. The tagmentation mixture was subsequently amplified for 12–18 cycles via Phusion with an MGI adapter via a DNA library kit (BioGenuineTech, Shanghai, China) and sequenced via DNBSEQ-T7 (MGI) with PE150 for 6G.

### 2.3 Genome assembly, taxonomy, and functional annotation

The preprocessing of MGI metagenomic sequences was executed via fastp, with parameters set to --length_required = 50, −-qualified_quality_phred = 15, −-unqualified_percent_limit = 40, and --n_base_limit = 10, for the purpose of filtering low-quality reads and trimming adapters. High-quality sequences were subsequently assembled via MEGAHIT (v1.2.9) (Li et al., 2016). Contigs exceeding 200 base pairs were selected for final annotation and gene prediction. The Burrows–Wheeler Alignment tool (BWA-mem2) was used for clean read alignment (Li and Durbin, 2009), followed by genome quality checks and calling counts for unigenes via checkm (Parks et al., 2015). Taxonomic categorization was accomplished with Kraken2 (Wood et al., 2019), and unigenes were subjected to homology searches against the Kyoto Encyclopedia of Genes and Genomes (KEGG) database. Functional annotations were derived from EggNOG analysis (Huerta-Cepas et al., 2017). DiTing was used for biogeochemical cycle analysis (Xue et al., 2021).

## 3 Results

### 3.1 Microbial community composition on the walls of the Ancient city

**Figure 6:**
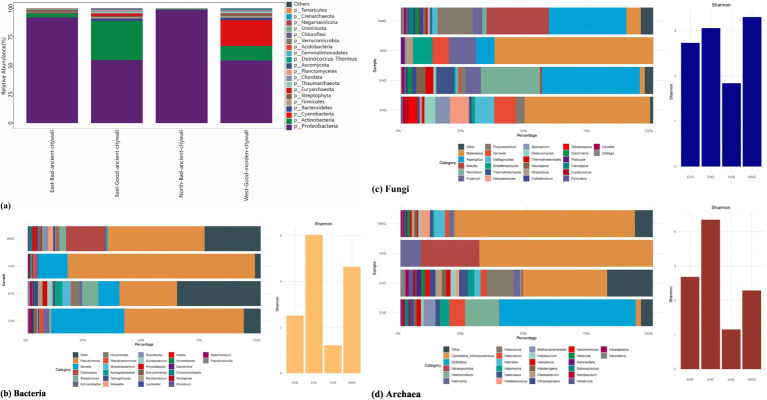
Genus level identification of archaeal, bacterial, and fungal communities, and alpha diversity from the Shou County ancient walls in China. a, Overall differences between walls. b–d, Abundance and Shannon diversity of bacteria, fungi, and archaea in EAB, EAG, NAB and WMG samples.

Using the MEGAHIT software suite, we processed and assembled the metagenomic sequences to assess the diversity and composition of the microbial community in the bricks of the Shou County ancient city wall. The predominant bacterial communities identified were Proteobacteria, Actinobacteria, Cyanobacteria, Bacteroidetes and Firmicutes, which collectively constituted more than 90 and 75% of the total microbiome at the phylum level, respectively (Figure 6a). Additional bacterial phyla detected in these communities in all samples in this study included Planctomycetes, Deinococcus-Thermus, Gemmatimonadetes, Acidobacteria (Kielak et al., 2016), Verrucomicrobia, Chloroflexi, and Tenericutes (Figure 6b). The sole fungal phylum present, Ascomycota, represented approximately 3% of the microbiome in structurally sound walls, whereas it comprised less than 0.1% of the microbiome in deteriorated walls (Figure 6a). In every sample analyzed, the phyla Euryarchaeota and Thaumarchaeota were present. Notably, the abundances of these phyla were greater in the ancient segments of the good wall than in the modern segments. Additionally, the phylum Crenarchaeota was exclusively identified in samples taken from the east wall. However, collectively, these archaeal phyla constituted a mere fraction, not exceeding 1% of the total microbial community (Figure 6a).

### 3.2 Microbial diversity on the Ancient city wall in Shou County

The taxonomic profiles of bacteria, archaea, and fungi at the genus level were extracted from the metagenomic datasets (Figures 6b–d). A phylogenetic investigation of the metagenomic sequences revealed a notably diverse bacterial microbiota colonizing Shou County’s edifices, with a range of 307 to 1,581 bacterial taxa. In contrast, archaeal and fungal taxa demonstrated more limited diversity, with the number of archaeal taxa ranging from 3 to 78 and the number of fungal taxa ranging from 7 to 44. The bacterial Shannon diversity indices for the EAB, EAG, NAB, and WMG samples were 2.50, 6.03, 1.21, and 4.63, respectively; for the archaeal samples, the indices were 2.68, 4.35, 1.15, and 2.29; and for the fungal samples, they were 2.73, 3.06, 1.84, and 3.30. The Shannon indices indicated that the bacterial populations at the well-maintained wall sites (EAG and WMG) presented greater diversity than did those at the deteriorated wall sites (EAB and NAB). Furthermore, the archaeal community exhibited greater diversity at ancient wall sites (EAG, EAB) than at more modern wall sites (WMG). However, in the case of fungal populations, the Shannon diversity metrics suggested that the ancient sites had lower diversity than the modern wall sites (WMG).

### 3.3 Differences in bray–Curtis distances and major microbial communities

**Figure 7:**
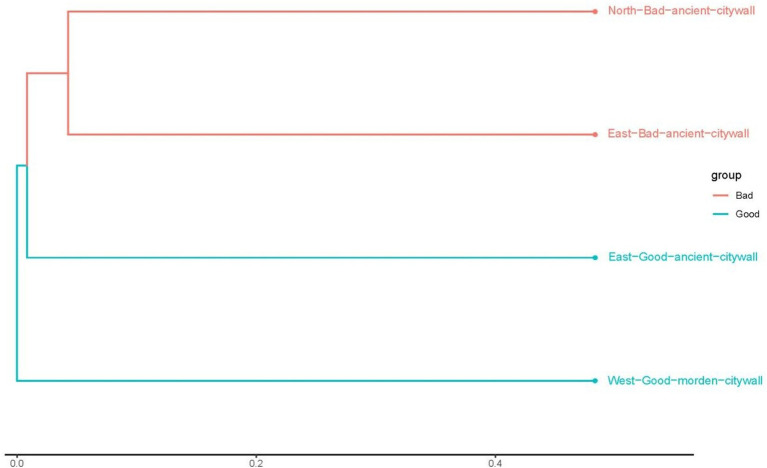
Bray–Curtis distances between the communities in the NAB, EAB, EAG, and WMG samples in Shou County. The black line and the boxes within each violin plot represent the median values and the 95% confidence intervals, respectively, and the whiskers in each violin plot represent the range. Different lower-case letters on top of the violin plots indicate a significant difference (*p* < 0.05) among other groups on the basis of Kruskal–Wallis multiple comparisons.

**Figure 8:**
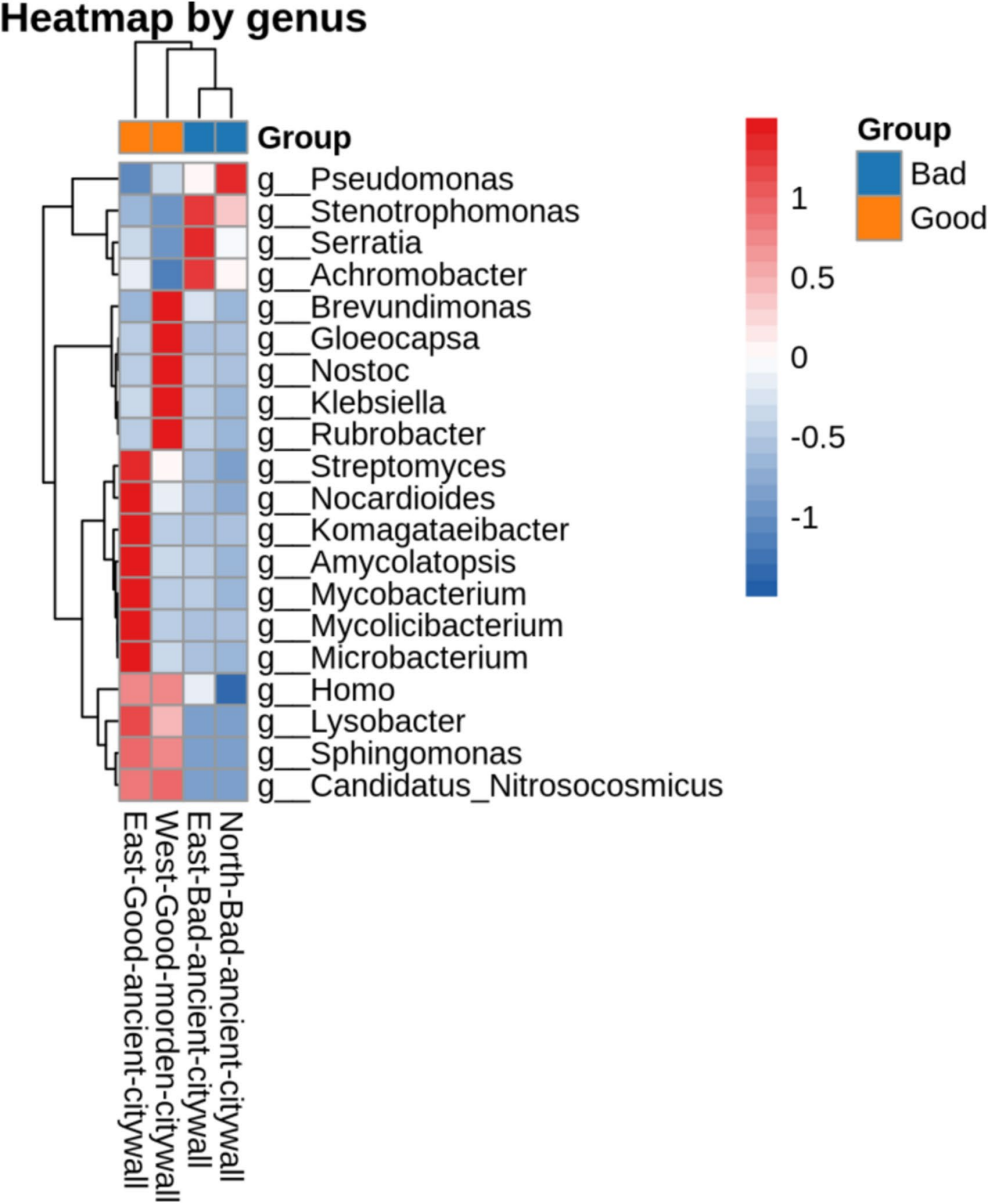
Heatmap of genus-level distributions in the NAB, EAB, EAG, and WMG samples.

To analyze the differences between ancient and modern walls and between bad and good walls at the genus level, the Bray–Curtis distances between samples determined on the basis of species composition are displayed in Figure 7. According to Bray–Curtis distances, the microbial compositions of bad walls are similar, whereas those of modern walls differ from those of ancient walls. The differences in the bacterial communities of all the samples are illustrated in Figure 8. The differential genera found in NAB and EAB were *Pseudomonas* and accounted for approximately 80 and 50%, respectively; in contrast, *Pseudomonas* accounted for less than 40% of the genera in the good walls (EAG and WMG). Interestingly, in EAB samples, *Serratia*, *Stenotrophomonas* and *Achromobacter* were significantly predominant in the bacterial community. *Lysobacter* and *Candidatus Nitrosocosmicus* accounted for approximately 1% of the bacteria in both the EAG and the MWG samples and accounted for a greater proportion of the bacteria in the bad wall. *Gloeocapsa* accounted for 16% of the bacteria in the EAG samples, whereas *Brevundimonas Nostoc*, *Klebsiella*, and *Rubrobacter* were more abundant. Evidence of human activity was detected on all walls, and the activity of these bacteria on good walls was greater than 0.1%.

### 3.4 Functions of biofilms on the bricks of the Ancient city wall in Shou County

**Figure 9:**
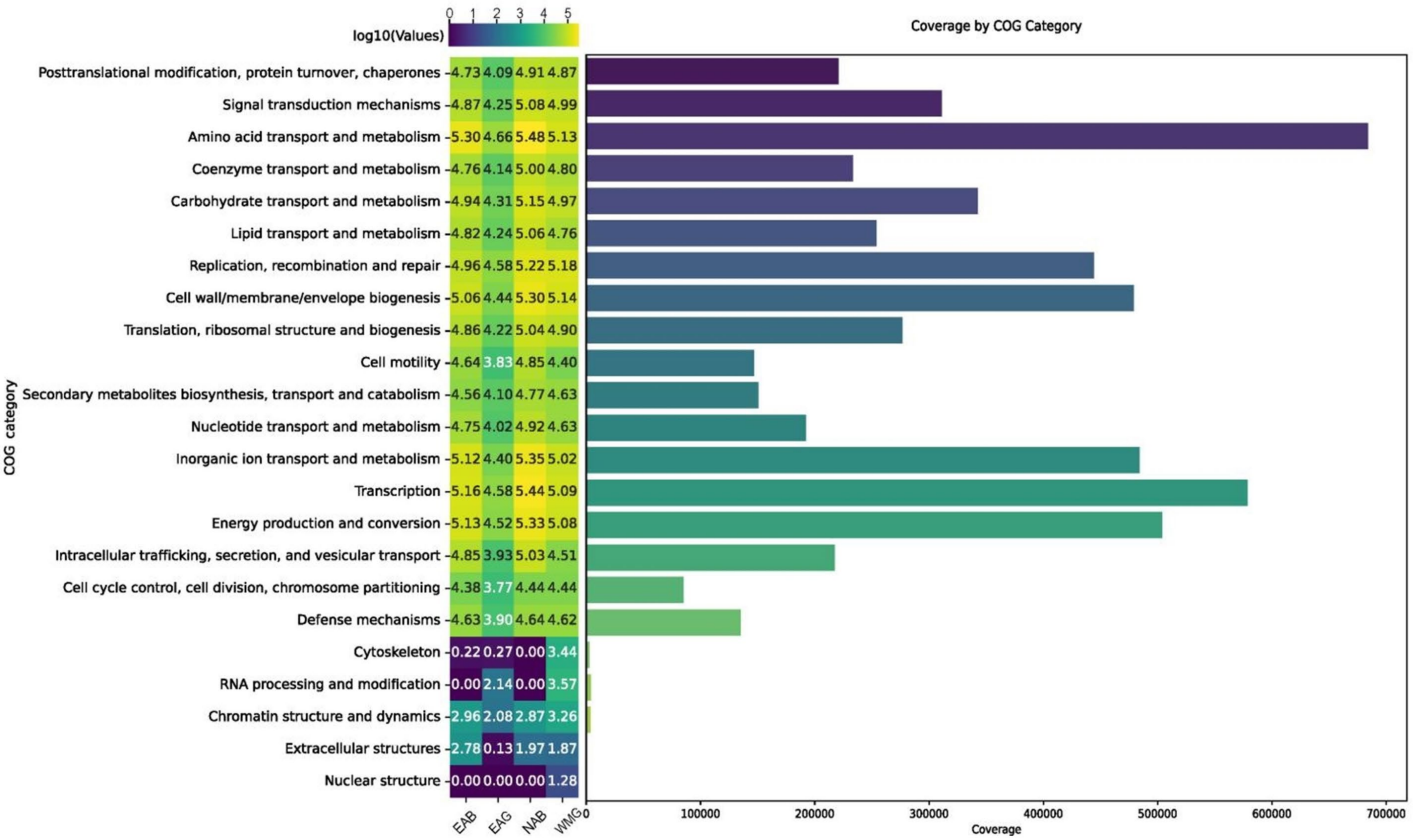
The ranks of COG functional categories within the brick microbiome determined at the metagenome level.

The biofilms of the county wall were annotated via EggNOG analysis and clustered by COG categories (Huerta-Cepas et al., 2017). The overall sequences obtained in this metagenomic dataset matched 24 COG functional categories, which were organized into the functional categories of metabolism, cellular processes and signaling, and information storage and processing (Figure 9; Supplementary Table S1). The majority of the sequences were associated with the COG category of transcription as well as amino acid transport and metabolism, and there were few hits in other categories, including chromatin structure and dynamics, nuclear structure, RNA processing and modification and cytoskeleton formation. In the good ancient walls, the sequence numbers were lowest in all COG categories, especially in metabolism and cellular processes, whereas those for transcription, energy production, and inorganic ion transport and metabolism increased in the bad walls. In particular, the energy metabolism and conversion functional categories ranked fourth in the microbiome and were significantly enriched in the NAB group.

### 3.5 Metabolic mapping of geomicrobiological cycles

The processes of carbon fixation, nitrogen transformation, and sulfur turnover constitute critical biogeochemical pathways for microbial energetics and the transmutation of substratum constituents, facilitating the sustenance of microbial communities on lithic substrates under a scant exogenous nutrient supply (Zanardini et al., 2019). Within this framework, an analysis of a metagenomic dataset revealed that 10.9% of the total sequence reads, corresponding to 15,989 annotated genes (Supplementary Table S1), aligned with 47 KEGG categories related to geomicrobiological cycles (refer to Appendix B). These data are instrumental in pinpointing the specific microbial taxa involved in facilitating geobiological processes related to energy harnessing and materials transformation, as elucidated by DiTing (Xue et al., 2021).

#### 3.5.1 Carbon cycling

**Figure 10:**
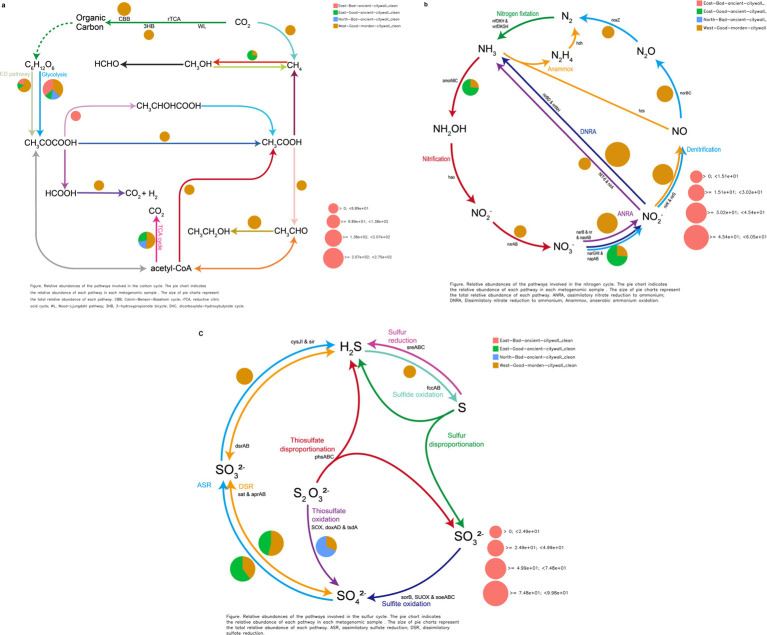
Geomicrobiological carbon, nitrogen, and sulfur cycling functions from DiTing. Pie charts representing the relative abundance of carbon (a), nitrogen (b), and sulfur (c) cycle-related pathways for three metagenomic samples. The normalized relative abundance was calculated by dividing the relative abundance of a pathway in an individual sample by the sum of the pathway’s relative abundance in all samples. The pie chart area reflects the relative abundance of the process according to the scale shown in pink. (a) CBB, Calvin–Benson–Bassham cycle; rTCA, reductive citric acid cycle; WL, Wood–Ljungdahl pathway; 3HB, 3-hydroxypropionate bicycle. (b) ANRA, assimilatory nitrate reduction to ammonia; DNRA, dissimilatory nitrate reduction to ammonia; Anammox, anaerobic ammonia oxidation. (c) ASR, assimilatory sulfate reduction; DSR, dissimilatory sulfate reduction. (a) CBB, Calvin–Benson–Bassham cycle; rTCA, reductive citric acid cycle; WL, Wood–Ljung–Dahl pathway. (b) ANRA, assimilatory nitrate reduction to ammonia; DNRA, dissimilatory nitrate reduction to ammonia; Anammox, anaerobic ammonia oxidation. (c) ASR, assimilatory sulfate reduction; DSR, dissimilatory sulfate reduction.

Functional annotation of sequences revealed the presence of genes involved in CO_2_ fixation and central metabolism in the microbiome. In the examined metagenomic dataset comprising 15,989 annotated genes, 455 (2.84%) and 403 (2.53%) were identified as being integral to carbon dioxide assimilation and fundamental metabolic processes, respectively, as delineated in Appendix A and mapped against the KEGG database (Supplementary Table S2). A more detailed overview of the annotated metagenomic reads grouped under the category of CO_2_ fixation pathways of prokaryotes revealed the dominance of sequences affiliated with the Calvin–Benson–Bassham cycle. The abundance of annotated metagenomic reads affiliated with the incomplete reductive citrate cycle was also determined. Other less abundant groups in the annotated metagenomic reads included the dicarboxylate–hydroxybutyrate cycle, reductive citric acid pathway, and 3-hyroxypropionate cycle. Sequences related to the CH_4_ oxidation pathway of methanotrophs and methanogenic pathways of methanogens were also detected (Figure 10a).

#### 3.5.2 Nitrogen cycling

Annotation of genomic sequences revealed a subset of genes involved in nitrogenous compound biotransformation within the microbial consortium. Among the 15,989 genes annotated and aligned with the KEGG database in this metagenomic compilation, 0.3% (48 genes) corresponded to nitrogen metabolism pathways. Notably, we identified specific enzymes characteristic of anaerobic oxidation of methane (AOM), such as ammonia monooxygenase (AMO), by detecting sequence variance in the amoA gene, an alpha subunit of AMO exclusive to Candidatus Nitrosocosmicus within the EAG samples. These are prevalent across both pristine and anthropogenically influenced biomes. Moreover, the napB gene, which is unique to *Serratia* within the EAG samples and is absent in deteriorated ancient masonry, is essential for the dissimilatory nitrate reduction process.

#### 3.5.3 Inorganic sulfur cycle

In the annotated sequence data, genomic analysis revealed a subset of genes, comprising 0.38% (60 out of 15,989) of the total genes, that were potentially involved in sulfur metabolic processes according to KEGG pathway correlations within this metagenomic dataset. Notably, within the NAB-specific *Pseudomonas* populations, the presence of the sulfur-oxidizing SoxY protein, which is known to facilitate the oxidation of thiosulfate to sulfuric acid through the SOX enzyme complex, was detected. Interestingly, sulfate adenylyltransferase, which is based on the sequence of cysD and sulfate adenylyltransferase subunit 2, was activated in all wall bacteria belonging to *Pseudomonas*, such as *Pseudomonas* sp. and *Pseudomonas fluorescens*, for dissimilatory sulfate reduction and assimilatory sulfate reduction. cysNC, cysN, ferredoxin, sat, and met3 were present in *Serratia* and *Pseudomonas* in WMG samples for dissimilatory sulfate reduction and assimilatory sulfate reduction.

## 4 Discussion

### 4.1 Microbial populations in brick metagenomes associated with the geoecological attributes of bricks

The Shannon diversity of the metagenomic dataset suggested that the bacterial communities of the Shou County walls were highly diverse, with lower diversity in the archaeal and fungal communities (Figure 6). The number of bacterial and archaeal genera identified on the walls is linked to geoecological characteristics, including the ability of the walls to withstand extreme environmental conditions, and these genera play essential roles in the geomicrobiological cycling of carbon, nitrogen, and sulfur. These include genera capable of autotrophic carbon fixation (e.g., *Phormidium* and *Rhodobacter*), genera driving the geomicrobiological nitrogen cycle (e.g., *Nitrososphaera* and *Novosphingobium*) and genera participating in the sulfur cycle (e.g., *Rhodovulum*). Notably, *Candidatus Nitrosocosmicus*, a member of the ammonia-oxidizing archaeal group in the nitrogen cycle, dominated the archaeal community on ancient walls but not in WMG samples (Figure 6b). Furthermore, a multitude of microbial genera (such as *Roseomonas* and *Microvirga*), which may have originated from fecal or external ecosystems associated with animal activities, were also identified in the microbial community of the walls, notably in good walls (Wu et al., 2023). Given the proximity of the sampling sites to cultural heritage and visitor areas that are managed by the government to protect ancient walls, it is speculated that the influence of plants and animals in and around the sites cannot be overlooked when the microbial composition of the wall microbiome is analyzed. Here, we found that the oxidation protein SoxZ, which is predicted to function in *Pseudomonas putida KT2440* and *Pseudomonas aeruginosa PAO1,* is significantly high in NAB samples, and the SoxR-reducing system protein RseC, which is predicted to function in *Serratia*, appears in GAB samples, indicating the influence of sulfur oxide on bad walls.

### 4.2 Geoecological influences on microbial diversity in Ancient city walls

The ancient city walls were constructed with glutinous rice lime mortar, clay and brick stone, and the brick material was relatively homogeneous. The detected changes in the microbial community composition of the walls are probably attributable to the geoecological influences and the level of human activities near the sampling locations. The use of glutinous rice lime mortar and brick stone may increase the amount of carbon available for glycolysis during the carbon cycle. The ancient city is located north of a national highway, where frequent traffic flow results in excessive emissions of vehicle exhaust, which are sources of sulfur and nitrogen; as a result, *Candidatus Nitrosocosmicus* was the core microbial agent for ammonia monooxygenase production for nitrogen cycling, and SoxY was enriched to produce sulfuric acid from thiosulfate, but this was not observed in the EAB samples. To the southwest of the area is the town of Yinxian, where Yin Xian firecrackers are traditionally crafted, yielding a large amount of sulfur-and nitrogen-containing products. Thus, the distribution of microbial members was strongly correlated with the geoecological characteristics of the sampling sites, and the enrichment of specific members of the wall microbiome was a response to the challenging environmental conditions of the ancient walls.

### 4.3 Metagenomes on the city wall bricks

On the basis of the metagenomic data in this study, it was determined that the microbial communities of the Shou County walls were largely dominated by *Actinobacteria*, *Proteobacteria*, and *Cyanobacteria*, all of which possess ecophysiological traits for colonization and community survival on bricks. Specifically, *Cyanobacteria* support the biofilm community and biofilm development through photosynthesis (Liu et al., 2020). *Actinobacteria* extend hyphal coverage over a large area to promote communities on bricks (Kusumi et al., 2011; Wu et al., 2022), and *Proteobacteria* contribute to biofilm development. Although these brick microbial communities contain different members, the dominance of these phyla in the brick microbiome is consistent with currently available information. Other bacterial phyla in brick microbial communities include *Deinococcus-Thermus* and *Bacteroidetes*, especially radiation-resistant *Deinococcus*, under high-UV irradiation conditions (Zanardini et al., 2019). In addition, many bacteria in the genera *Actinobacteria* and *Cyanobacteria* are capable of producing pigments for protection against UV light and can propagate under oligotrophic conditions on brick surfaces (Berg, 2011; Gtari et al., 2012; Egas et al., 2014; Hezbri et al., 2015). Since the surfaces of modern walls are oligotrophic, the input of organic nutrients from photosynthesis or atmospheric carbon sequestration plays a vital role in the initiation of the microbiome and modification of the wall surface. In this study, *Cyanobacteria* were found to be more abundant on the modern wall (WMG) than on the ancient glutinous rice paste walls (EAG, EAB, and NAB). Autotrophs play a key role in generating biomass on brick surfaces, thereby creating an organic nutrient-rich environment for the proliferation of heterotrophic microorganisms. This process leads to the development of diverse microbial biofilms on the surface of brick walls, posing risks of physical damage (Liu et al., 2020). This is because biofilms containing diverse microorganisms are present, and the microorganisms have the capacity to generate various metabolites, including extracellular polymeric substances (EPSs), inorganic acids, and organic acids. These EPSs, which are primarily composed of polysaccharides, have the ability to capture airborne particulates and environmental pollutants, intensifying their detrimental impact on biofilms. The ecophysiological characteristics of the microorganisms in the complex microbial community in this study were delineated via the COG database (Tatusov et al., 1997). The abundance profiles of the metagenomic COG functions are summarized and ranked in Figure 5. Differences were observed in the distribution patterns of the COG categories between good and bad walls in this study. The differences in the descriptions of both the taxonomic structure profiling and the biochemical metabolic functions of the community revealed that the metagenome reflects the basic needs of the microbiome for survival under disturbances (Figure 9). The ratio of COG functional categories obtained from the metagenomes could be used to reveal the most important biochemical functions of the community. According to the results of this study, the most important biochemical function of the microbiome on Shou County walls is energy production and conversion (Figure 9).

### 4.4 Geomicrobiological biochemical reactions and metabolically active microbial participants

The basic and essential biochemical processes active in brick microbial communities are energy metabolism, which includes carbon sequestration and nitrogen and sulfur cycling and transformation (Liu et al., 2020; Zanardini et al., 2019; Gorbushina, 2007). On the basis of a previous study, it was found that the geomicrobiological cycle in the deteriorated sand bricks of the ancient city wall of Shou County can be mapped by matching the DNA sequences of the environmental metagenome with those in the KEGG database (Kanehisa et al., 2014). Carbon dioxide serves as the primary source of carbon for epilithic biofilms because of the limited availability of organic carbon on bricks. As a result, phototrophs such as cyanobacteria and algae, along with certain chemolithotrophs such as nitrifiers and sulfur-oxidizing bacteria, play a key role in converting CO_2_ into organic compounds to support subsequent biocolonizers (Sand and Bock, 1991). Here, all known CO_2_ fixation pathways were identified except for the reductive acetyl-CoA pathway. On the basis of DiTing analysis combined with eggNOG analysis, we found that *Serratia* and *Pseudomonas* are core microbes that play crucial roles in the complete carbon cycle chain, which includes carbon fixation pathways involving fbaA, fbp, and gapA in the Calvin–Benson–Bassham cycle (CBB); MTHFR in the Wood–Ljung–Dahl pathway (WL); accB, bccP and accA in the 3-hydroxypropionate bicycle (3HB); IDH1, IDH2, ppc, sdhC and frdC in the reductive citrate cycle (rTCA); central metabolism involving IDH1 and IDH2 in the TCA cycle; and pckA, GPI, gpmI and ENO in gluconeogenesis and glycolysis (Supplementary Table S2).

Active glycolysis, the ED pathway and the absence of the TCA cycle are significant factors affecting the state of the eastern ancient city walls, whereas these factors do not affect the EAG, NAB or WMG wall sections. Microorganisms, especially certain bacteria, contribute to carbon fixation through processes such as photosynthesis. During photosynthesis, autotrophic microorganisms convert inorganic carbon (usually carbon dioxide) into organic carbon compounds. Moreover, microorganisms breakdown complex organic compounds into simpler forms, releasing carbon dioxide into the atmosphere. A comprehensive analysis of the annotated metagenomic reads revealed that a significant proportion of the sequences fell under the category of CO_2_ fixation pathways of prokaryotes. Further examination of these reads revealed a dominant presence of sequences associated with glycolysis and the tricarboxylic acid (TCA) cycle. The annotated metagenomic reads also encompassed less prevalent groups, such as the Calvin–Benson–Bassham cycle, the 3-hydroxypropionate bicycle, and the reductive citric acid route. A comprehensive analysis of the annotated metagenomic reads, which were categorized under the CO_2_ fixation pathways of prokaryotes, revealed that the sequences predominantly belonged to the pyruvic acid to lactic acid or acetic acid pathway. Furthermore, the ED pathway was detected, which results in the generation of a large quantity of acids that expedite the process of corrosion on various building surfaces. For culture heritage site protection, the ED pathway could be utilized as a marker for biodeterioration, for example, via the analysis of pyruvate, lactic acid or acetic acid. Inhibitors of 2-dehydro-3-deoxyphosphogluconate aldolase, which limits the ED pathway, could be used for the protection of cultural heritage sites (Figure 9a).

For almost all the transformation reaction steps in the geomicrobiological nitrogen cycle, in EAG sections, the genes responsible for nitrification in *Candidatus nitrosocosmicus*, which have been widely found in natural and engineered environments, were retrieved from our metagenome dataset. These included AOM-specific functional enzymes, such as ammonia monooxygenase (AMO), which were determined on the basis of sequence discrepancies in the ammonia monooxygenase subunit alpha (amoA). napB in *Serratia* played an important role in dissimilatory nitrate reduction only in EAG sections and not in the bad ancient wall sections, leading to acid corrosion of the ancient wall. A high number of annotated sequences associated with the KEGG functional pathway for NO_3_^−^ and NO_2_^−^ transformation and a pathway associated with the conversion of NO_2_^−^ to NH_4_^+^ were detected on good walls, which demonstrated that dissimilatory nitrate reduction to ammonium (DNRA) likely occurs in WMG wall sections, suggesting that neutralization occurs on the good wall (Figure 10b). Recently, internal nitrogen recycling involving DNRA, ammonia oxidation, nitrification, and comammox reactions was shown to promote the conservation of available nitrogen for the sequestration and immobilization of inorganic C onto bricks through the simultaneous accumulation of biomass and the initiation of biodeterioration (Ding et al., 2020).

In this analysis, numerous annotated genes were retrieved, which were affiliated with the functional genes involved in the conversion of sulfate to sulfite via cysD for assimilatory sulfate reduction and dissimilatory sulfate reduction and then to sulfide via sat and met3 in Pseudomonas on good walls, suggesting the presence of microbially mediated assimilatory sulfate reduction pathways on the surface of these good walls (Figure 10c; Supplementary Table S1). Generally, SO_4_^2−^ reduction occurs through assimilatory and/or dissimilatory pathways (Ding et al., 2020). The sulfur assimilatory reduction pathway is present in a relatively wide range of microbial genera and results in the production of reduced sulfur compounds that are used primarily in the biosynthesis of sulfur-containing amino acids and biomass without the release of sulfides. In the dissimilatory pathway, which is limited to obligatory anaerobic bacteria and archaeal lineages, SO_4_^2−^ (or sulfur) is the terminal electron acceptor of the respiratory chain and is reduced to inorganic sulfide (H_2_S) (Grein et al., 2013). Surprisingly, annotated sequences affiliated with the SOX (sulfur-oxidizing) system were found in these metagenome datasets only in the NAB wall, indicating that acid corrosion originated from vehicle exhaust from the North National Road.

## 5 Conclusion

### 5.1 Research findings

The metagenomic analysis conducted in this study provides insights into the microbial characteristics of ancient walls in Shou County. Taxonomic annotation of the metagenomes from different sampling sites suggested the presence of a stable core microbial population associated with the geoecological attributes of the walls, e.g., thiosulfate oxidation by the SOX complex in the NAB wall. Moreover, site-specific analysis of the microbiome revealed that these brick metagenomes can reflect site-specific ecological signatures. The taxon distribution and metabolic functional distribution patterns as determined by metagenomic analysis clearly differed among different wall sections. Physiological functions related to “energy production and conversion” were most critical and were found to be metabolically active in the brick microbiome, as revealed by metagenomic analysis. In summary, this study revealed how the microbiome may contribute to self-supporting symbioses to improve the growth and fitness of microbes in these ancient walls.

The research highlights include mainly the following. (1) The well-preserved city wall is characterized by the nitrogen cycle processes of ammonia oxidation and nitrite reduction to nitrate, which indicates that nitrate may play a role in the weathering process of the city wall. (2) The proximity of the walls to the national highway causes direct and severe biological weathering in the northern city wall due to vehicle exhaust emissions, as evidenced by the significant enrichment of SoxY in the metagenomic data. This also suggests that the conversion of thiosulfate to sulfate leads to severe biocorrosion of the walls. (3) In terms of protection of the city wall from bacteria, the absence of active glycolysis, the ED pathway, and the TCA cycle are significant factors contributing to the destruction of the eastern city wall.

### 5.2 Limitations and prospects

Biotechnological advances provide scientific and technical guidance for protecting architectural heritage sites and offer new ways to promote method development. At present, there are few interdisciplinary studies on architectural heritage site protection and biotechnology. This research has several flaws and limitations. For example, (1) in the realm of biotechnology, researchers may struggle to swiftly and thoroughly identify optimal outcomes through experiments, frequently yielding unexpected outcomes. Newly developed biological inhibitors may not necessarily play a beneficial role. (2) From the standpoint of safeguarding cultural heritage sites, policies or regulations might prohibit the study of some rare and significant cultural assets or their use in biotechnological experiments. (3) From the perspective of experimental results, researchers currently lack a complete process for communicating test results and proposing corresponding strategies due to the difficulties associated with interdisciplinary studies and the lack of relevant research.

However, considering the importance of architectural heritage site protection, there is potential for further advances. (1) We used the NGS method to determine the composition and function of the metagenome and microbial community. Compared with 16S rRNA analysis, the NGS method can be used to better measure the functional genes in bacteria, with a focus on the sulfate cycle associated with frequent residential activities and the sulfuric acid produced by automobile exhaust. The ancient site at Shou County has existed for thousands of years, and people have lived at the site for a long time. Frequent human activities have caused serious microbial erosion to the architectural heritage site, making site protection more difficult. These results will prompt area managers to pay attention to the issue of site corrosion, protect the architectural heritage site, and propose more scientifically sound and reasonable suggestions during future transportation planning changes.

(2) Advanced microbial analysis technology can help cultural heritage site managers to determine the microecological environment of historical material relics. Mining and using advanced biotechnological data from metagenomics, carefully monitoring and determining the kinds of microorganisms or bacterial communities that are present and identifying where they are located, finding the core genes associated with elemental cycles, and creating targeted inhibitors against harmful bacteria or proteins will have a highly positive effect on the long-term environmental protection and preservation of material cultural heritage sites.

(3) Although this research model is still in its early stages, we can confidently predict that in the future, professionals in the fields of biology will assist cultural heritage site managers in achieving targeted and precise protection of cultural relics through the use of microbial inhibition technology. This will further guide the protection practices of numerous cultural heritage sites in other parts of China and provide a useful supplement to research on world cultural heritage site protection.

(4) The ideas, methods, and viewpoints of this study can serve as references for related research and are highly important for the protection of diversity in world culture.

Therefore, our hope for the future is as follows.

1. We will establish and improve systems for protecting cultural heritage sites and develop systems for repairing and inspecting architectural heritage sites. (a) China’s current systems and regulations on cultural heritage site protection still have much room for improvement. The maintenance and repair levels of architectural cultural heritage sites are nonuniform. There is insufficient oversight to monitor microbial damage to architectural cultural heritage sites, and there are no special laws or regulations to guide and constrain damage. Therefore, it is necessary to start at the legal level and establish a sound cultural heritage protection system so there is a legal basis for preventing microbial corrosion in architectural cultural heritage sites. (b) Currently, in China, repair work is being carried out in an orderly manner at architectural cultural heritage sites. However, there are issues such as the uneven regional distributions of funds, technology, and manpower, which lead to delays in repair work. Microorganisms have a long-lasting, profound, and irreversible impact on architectural cultural heritage sites, making it crucial to prepare for unforeseen circumstances. It is necessary to increase funding and establish relevant inspection and repair systems in advance to discover and solve problems in an expedient manner to protect cultural heritage sites more efficiently.
2. We will develop scientifically sound microbial control strategies for cultural heritage sites. In China, few people have approached cultural heritage site protection from a microbial perspective, and scientifically sound methods are urgently needed. Advanced biotechnological methods should be applied to establish effective microbial control measures for cultural heritage sites, which will further guide and assist in cultural heritage site protection work. (a) Quorum quenching-related preparations suitable for architectural cultural heritage sites should be developed. As the market for antimicrobial agents and disinfectants continues to grow, the utility of their application is becoming more unclear. Many studies have shown that broad-spectrum fungicides have certain negative effects on humans and the environment. The lack of active glycolysis, the EMP pathway, and the TCA cycle were all important factors in the fall of the east city wall. The use of broad-spectrum fungicides stops related carbon cycle metabolism, which could cause the same kind of damage to the east ancient city wall. Because of this, it is very important to use metagenomics to identify the most common bacterial communities and then develop ways to prevent these communities from damaging structures. This article can serve as a reference for related research. (b) Research and development should be conducted on the development of sustainable antifouling agents for building materials: Green and sustainable antifouling solutions should be sought for building materials, especially in developing promising quorum quenching (QQ) compounds. In this study, we investigated ancient city walls and discovered that they were rich in SOXY damage, which may have been caused by *Rhodovulum sulfidophilum*. Therefore, we can target the *Rhodovulum* genus with preparations related to group interference.

## Data Availability

The source genome data related to this article are submitted to the public database National Library of Medicine: https://www.ncbi.nlm.nih.gov/ (BioProject: PRJNA1104593).
