# Supplementary material for: Metagenomic study of the microbiome and key geochemical potentials associated with architectural heritage sites: a case study of the Song Dynasty city wall in Shou County, China
Source: Front Microbiol. 2024 Oct 25;15:1453430. doi: 10.3389/fmicb.2024.1453430 (PMC11543536; doi:10.3389/fmicb.2024.1453430)
Supplement: Supplementary file 2 [file Data_Sheet_1.docx]

Metagenomic Study of the Microbiome and Key Geochemical Potentials Associated with Architectural Heritage Sites: A Case Study of the Song Dynasty City Wall in Shou County, China

Mingyi Zhao ^1^† , Yanyu Li ^1^† , Huanhuan Chen ^1^† , Yile Chen ^1^† , Liang Zheng ^1^ , Yue Wu ^2^ , Kang Wang ^3^ , Zhao Pan ^4,^* , Tao Yu ^5,^* , Tao Wang ^6,^*

^1^ Faculty of Humanities and Arts, Macau University of Science and Technology. Avenida Wai Long, Taipa, Macau 999078, China

^2^ Shanghai Biogenuinetech Co., Ltd. Room 304, Building 4, Lane 58 Zhanling Rd, Pudong New District, 200137, Shanghai, China

^3^ College of Life Sciences, Qingdao University. No.308 Ningxia Road, Qingdao 266000, Shandong Province, China

^4^ School of Art & Design, Shandong Jiaotong University. No.5001 Haitang Road, Changqing University Science and Technology Park, Jinan 250300, Shandong Province, China

^5^ Institutes for Translational Medicine, Qingdao University. No.308 Ningxia Road, Qingdao 266000, Shandong Province, China

^6^ The Affiliated hospital of Qingdao University, No. 16 Jiangsu Road, Qingdao 266000, Shandong Province, China

^†^ These authors have contributed equally to this work and share first authorship.

*** Correspondence:**

panzhao2024@163.com (Zhao Pan); qumea0532@163.com (Tao Yu); wangtao59@qdu.edu.cn (Tao Wang)

**Appendix A: Climate analysis map**

The following is a detailed climate analysis chart of Shou County:

**
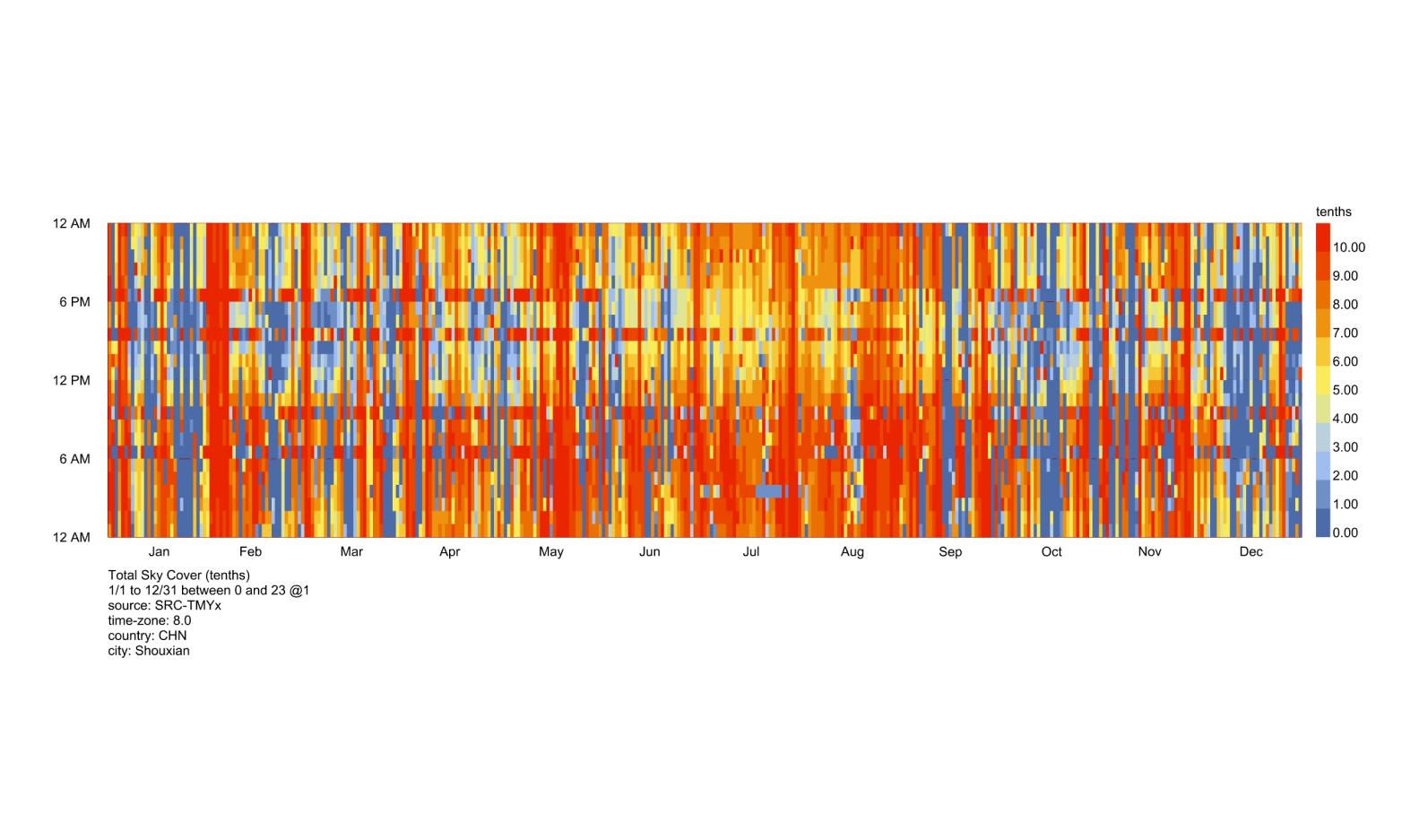
**

(1) Total sky cover

**
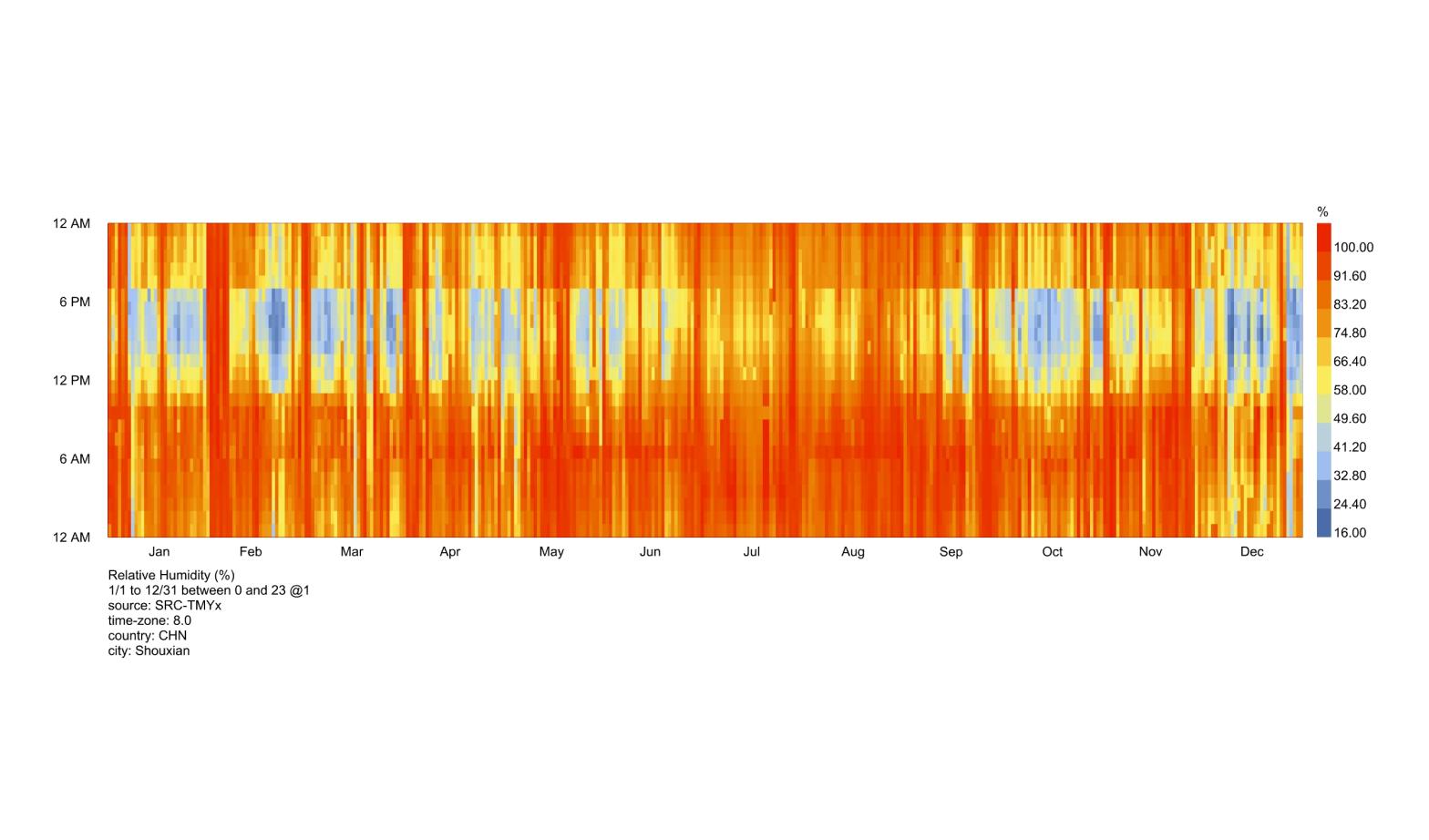
**

(2) Relative humidity (%)


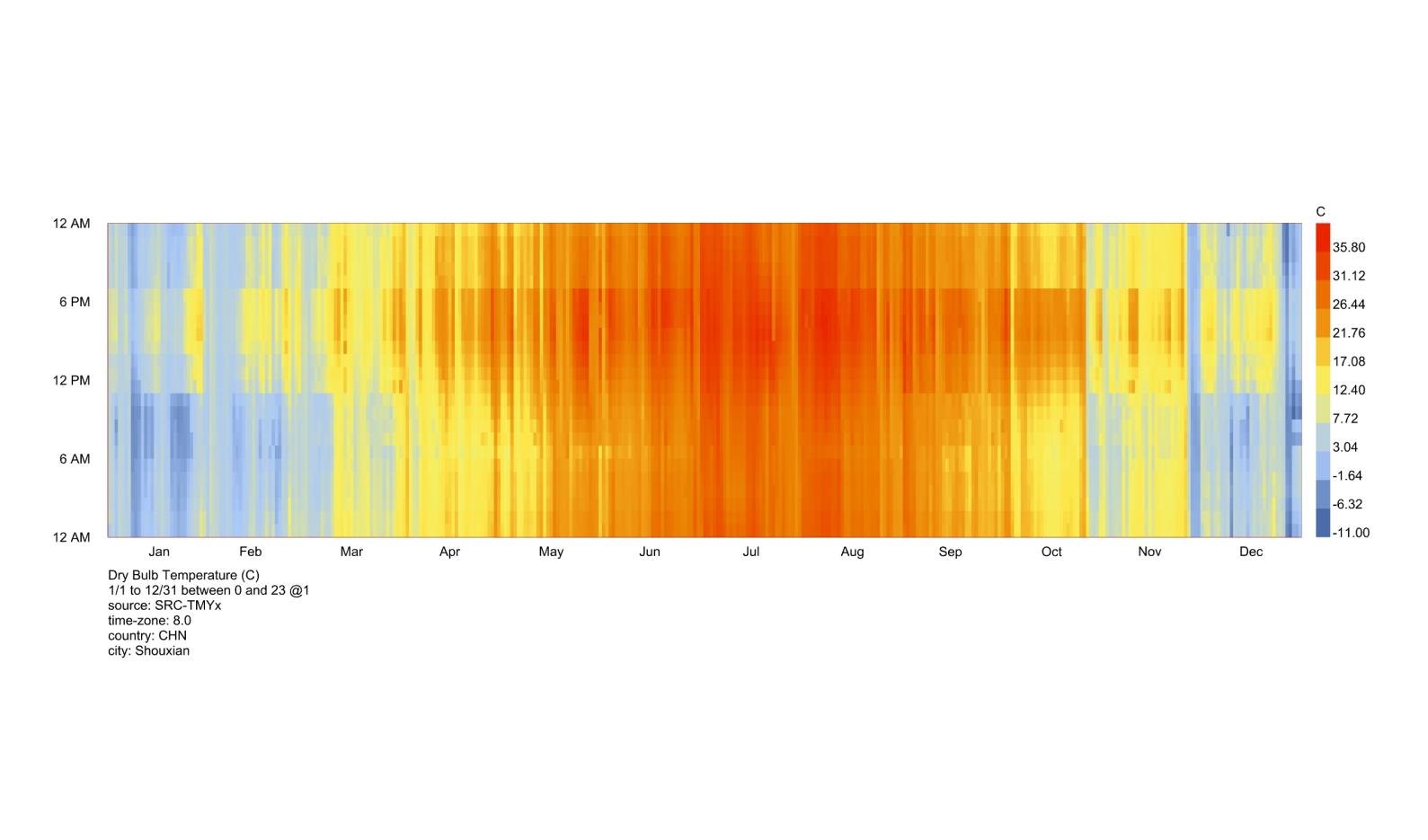


(3) Dry bulb temperature (℃)

**
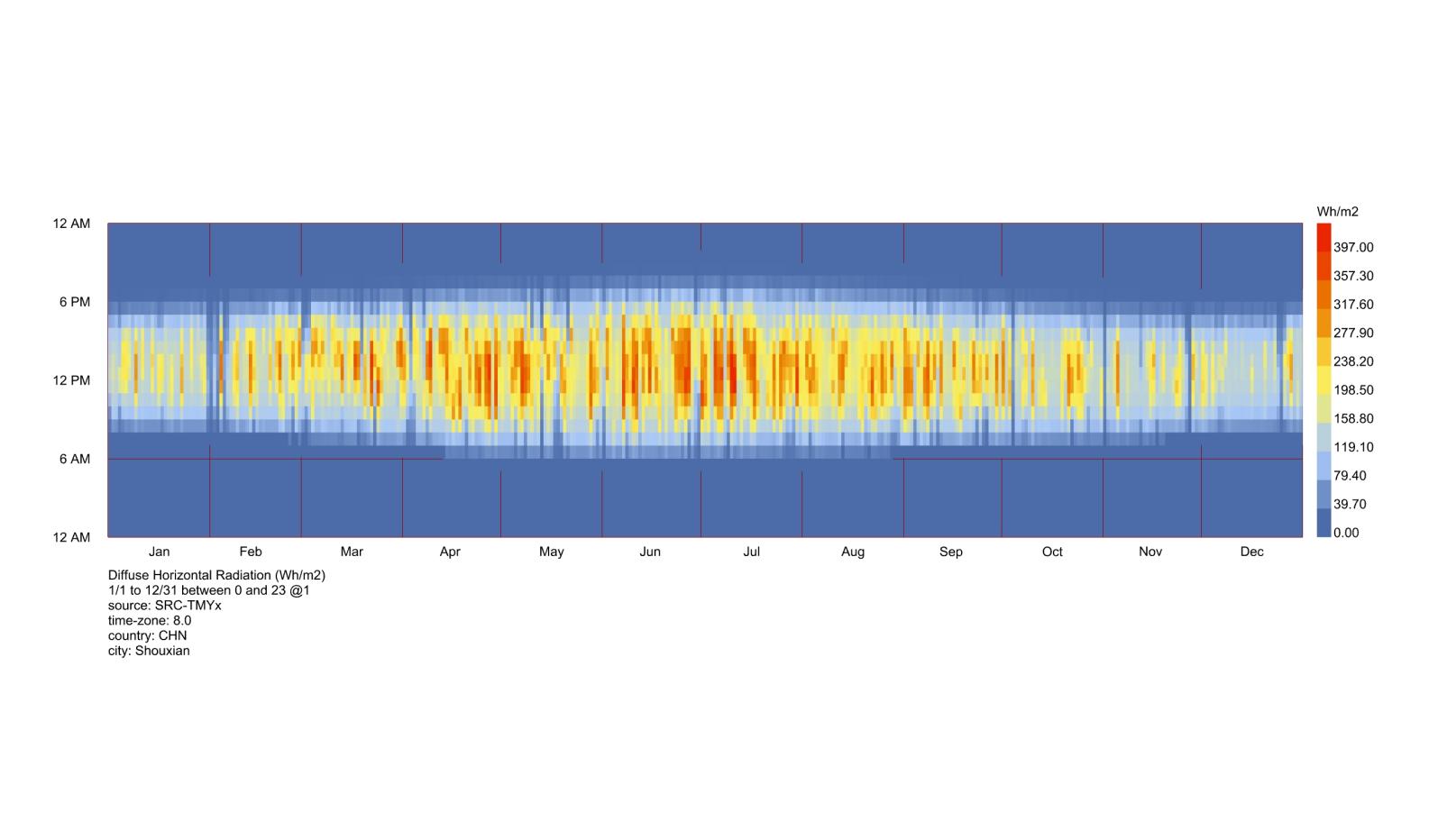
**

(4) Diffuse horizontal radiation (Wh/m2)


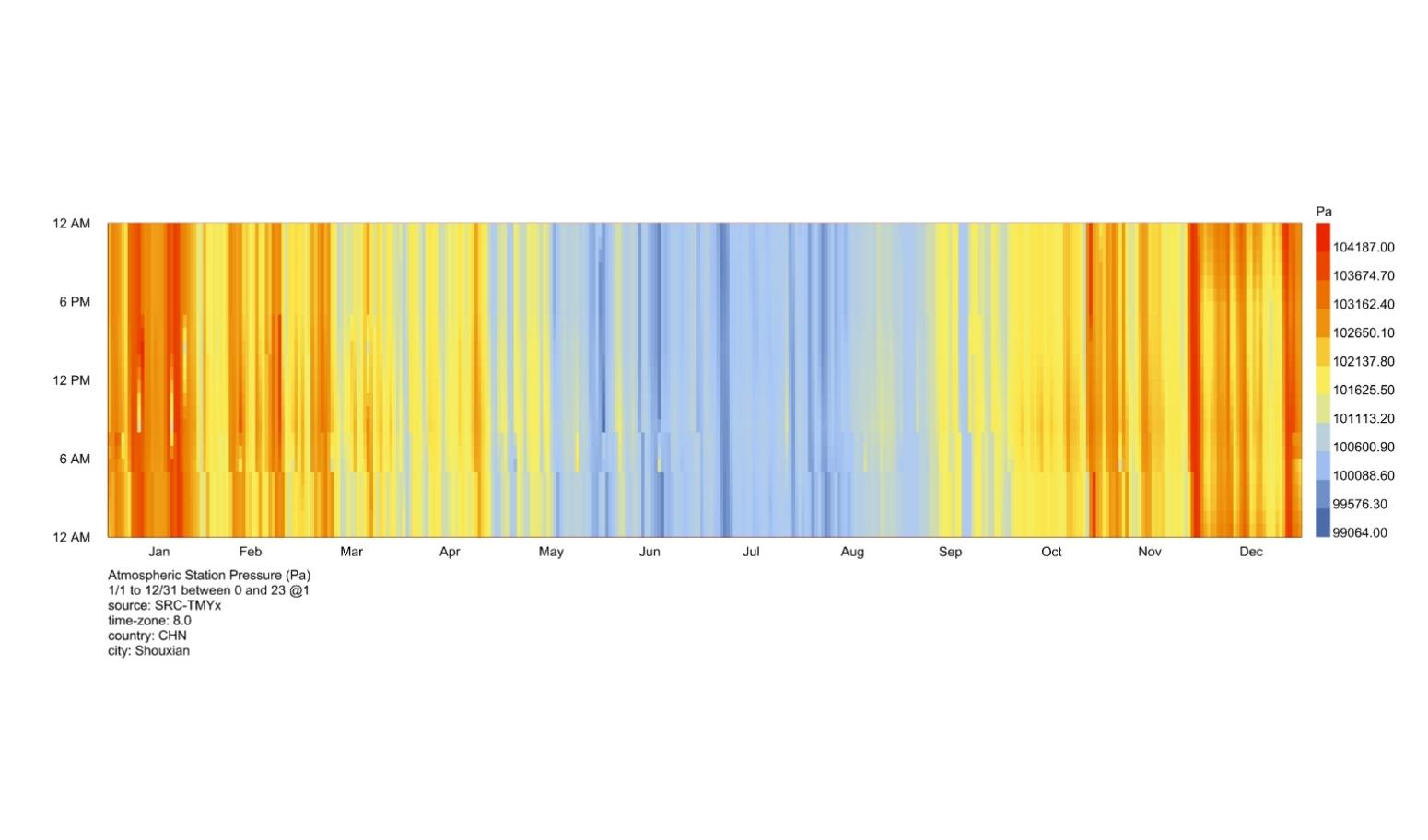


(5) Atmospheric Station Pressure (Pa)


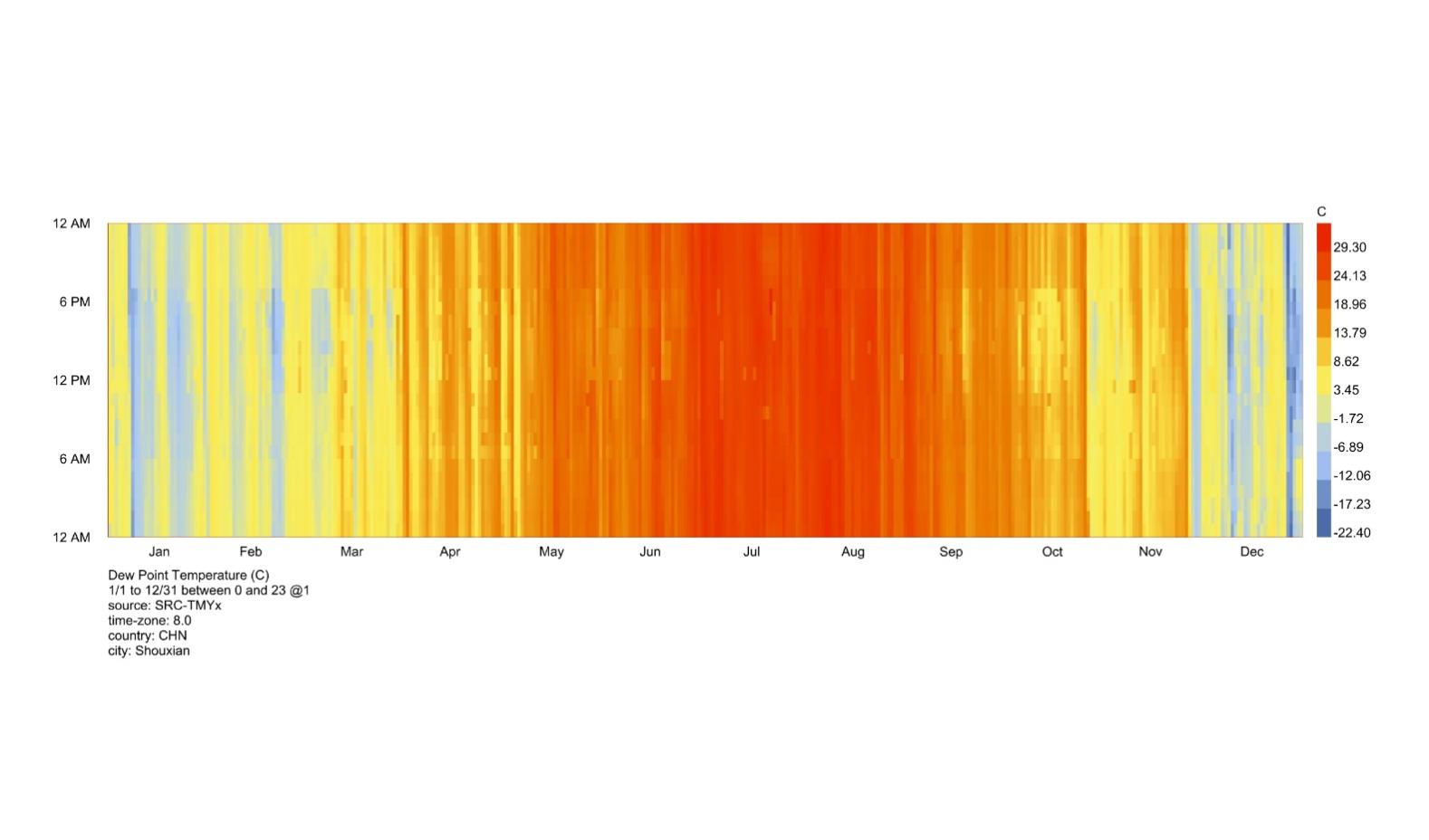


(6) Dew point temperature (C)


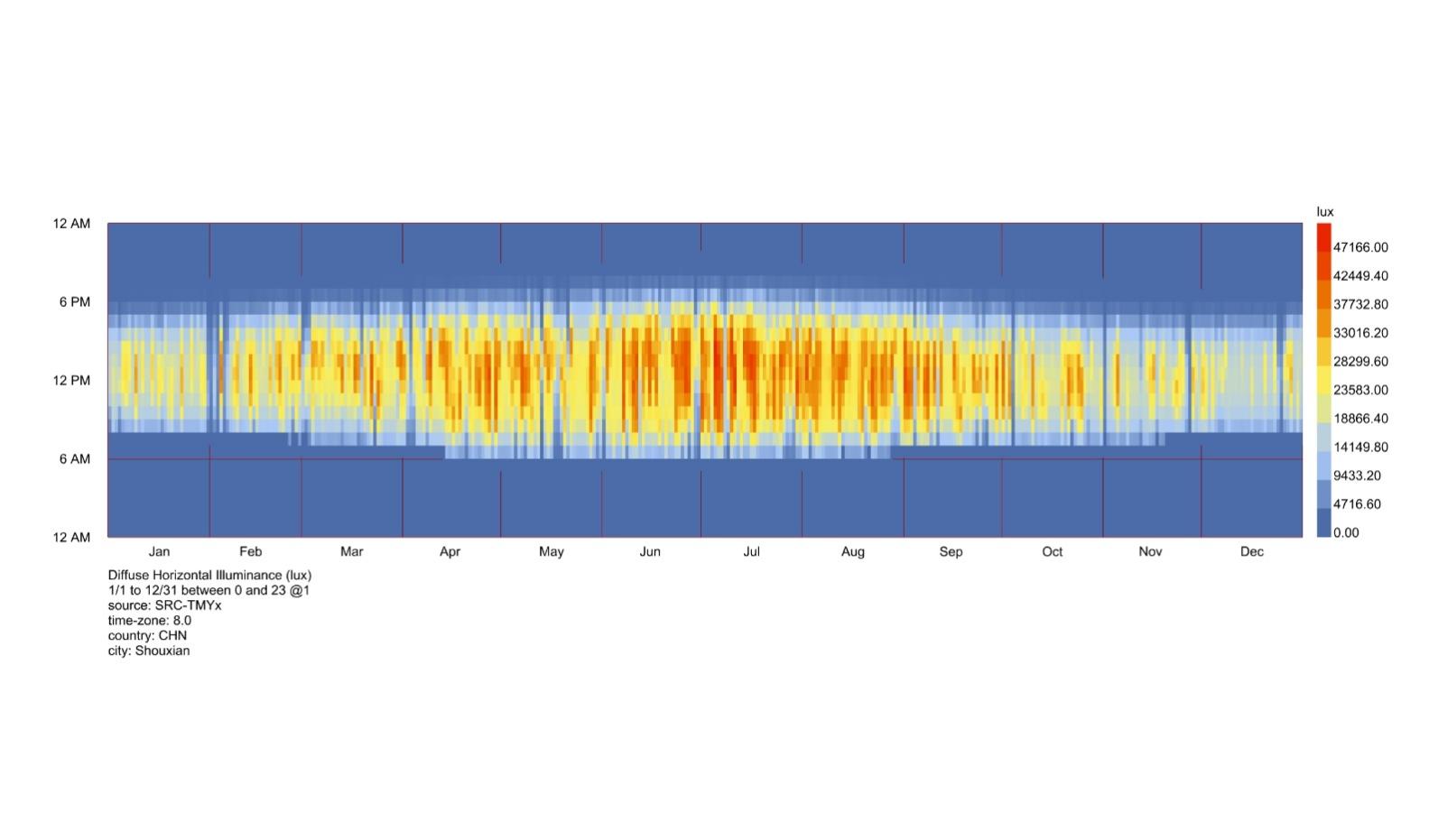


(7) Diffuse horizontal illuminance (lux)


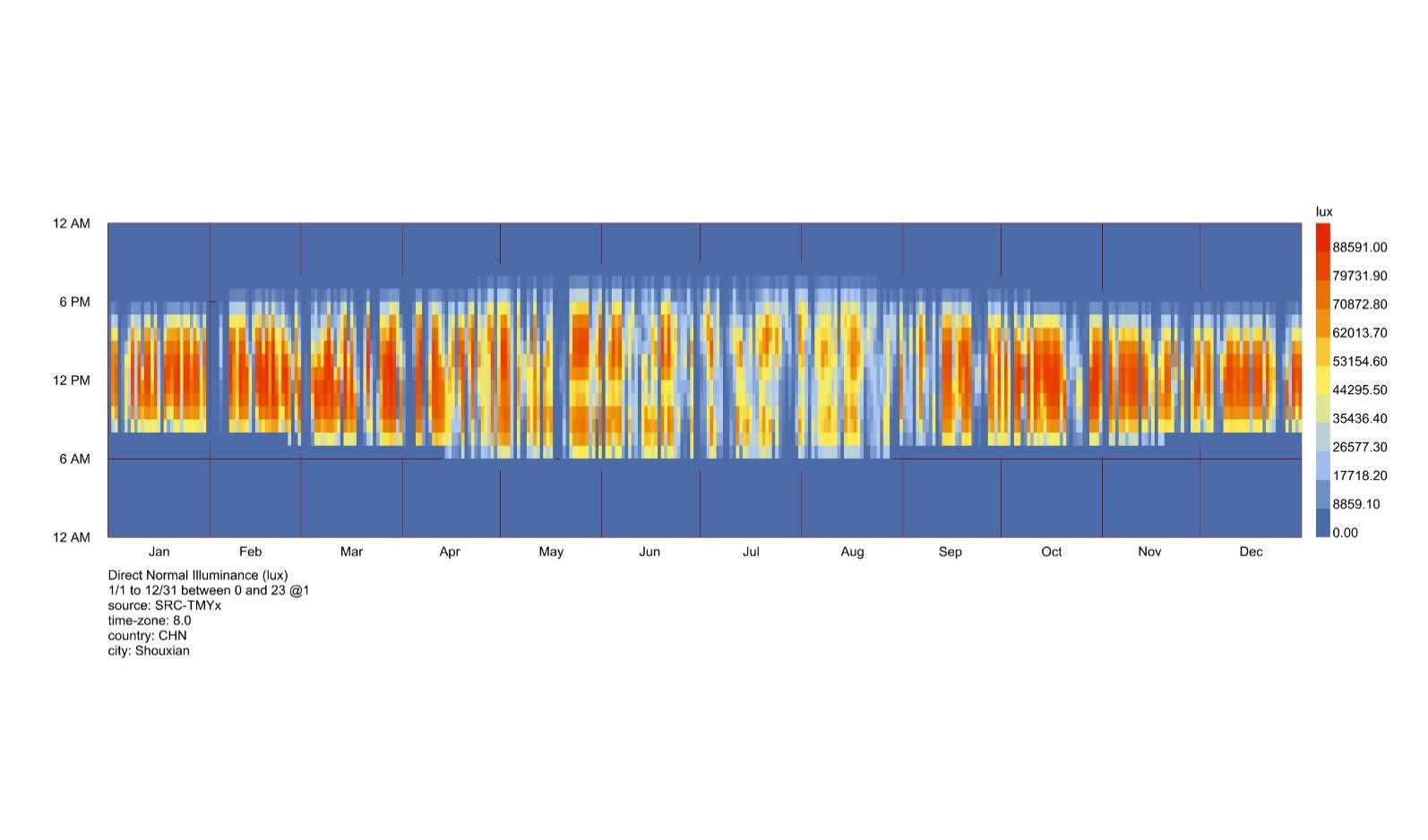


(8) Direct normal illuminance (lux)


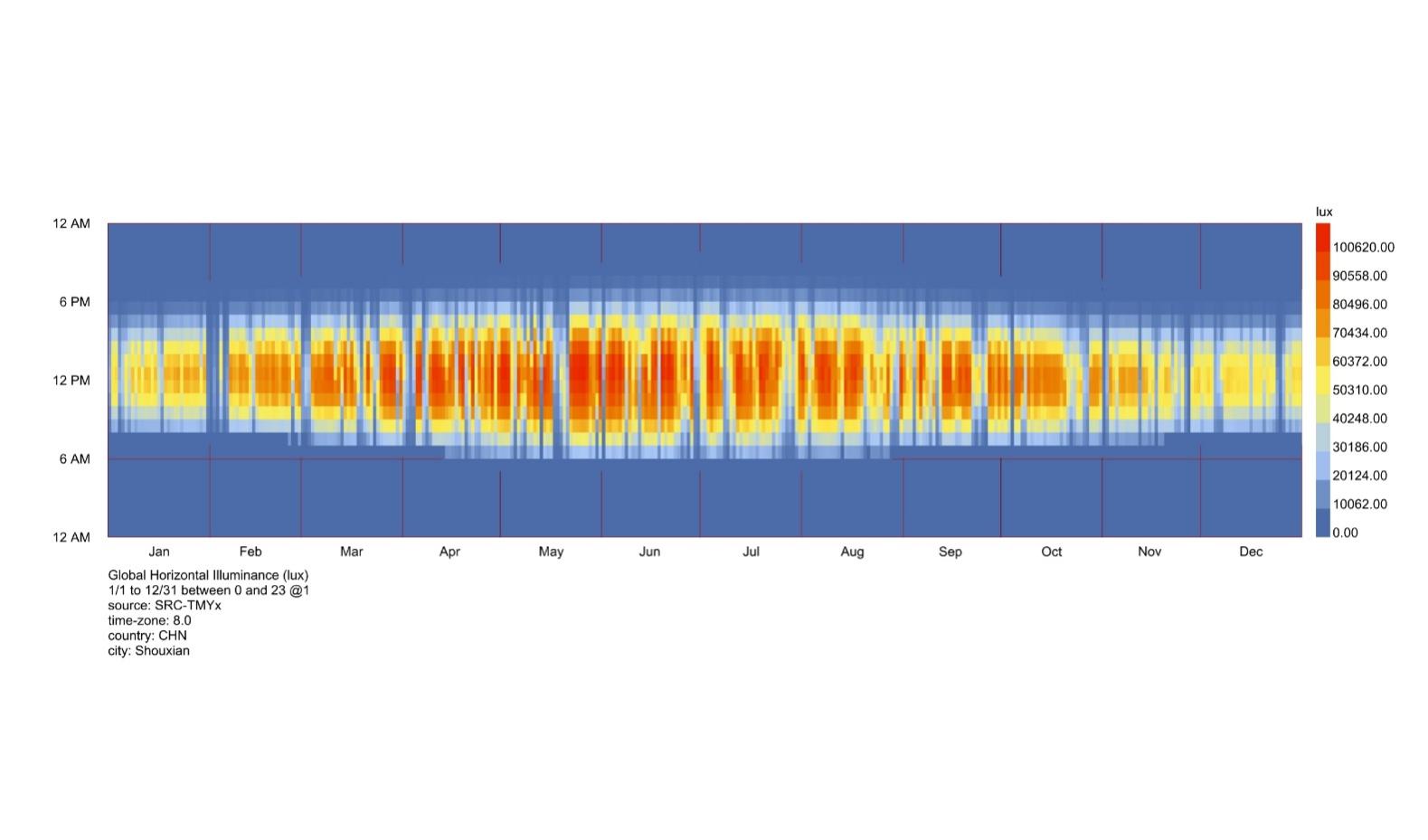


(9) Global horizontal illuminance (lux)


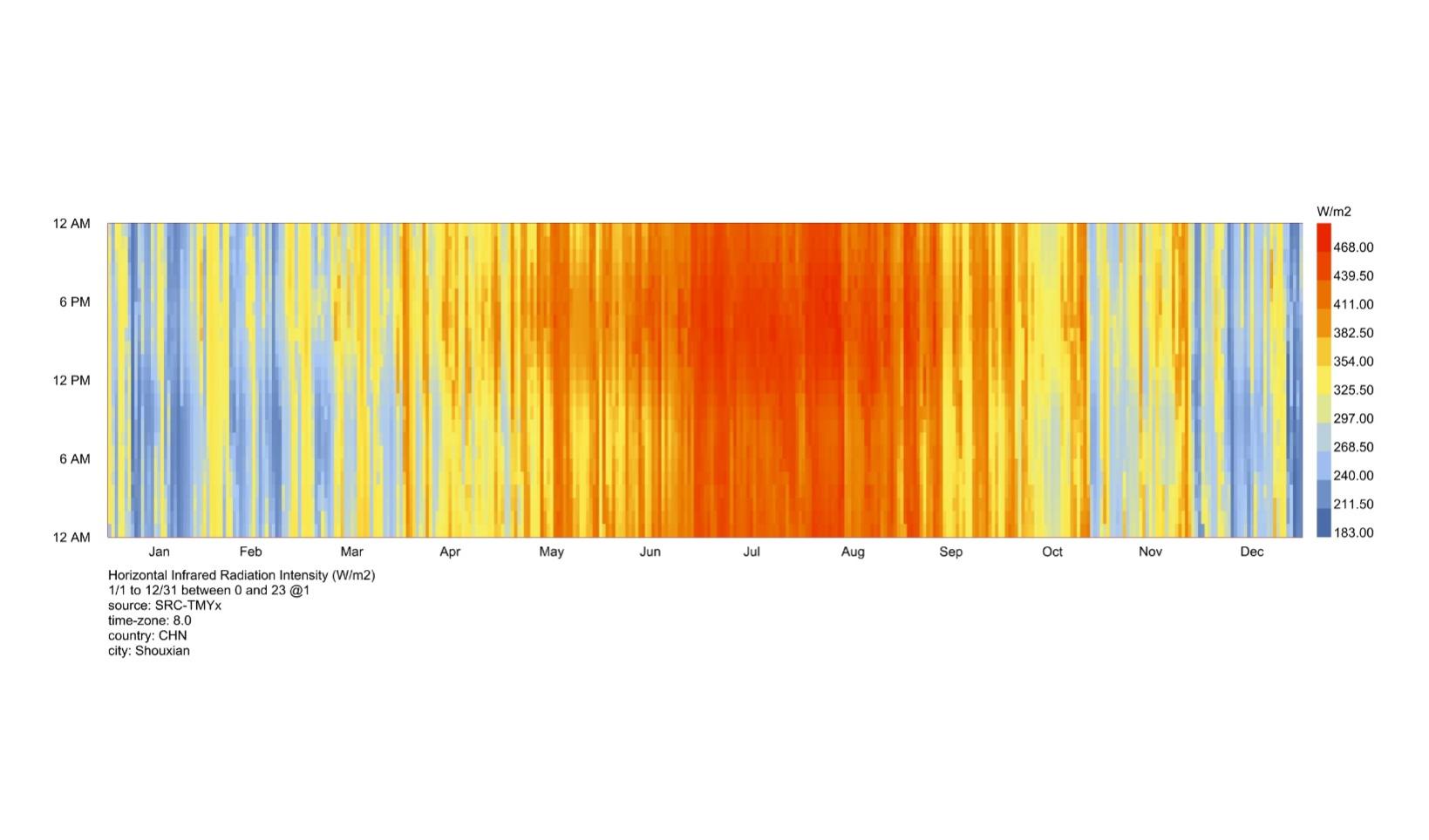


(10) Horizontal Infrared Radiation Intensity (Wh/m2)


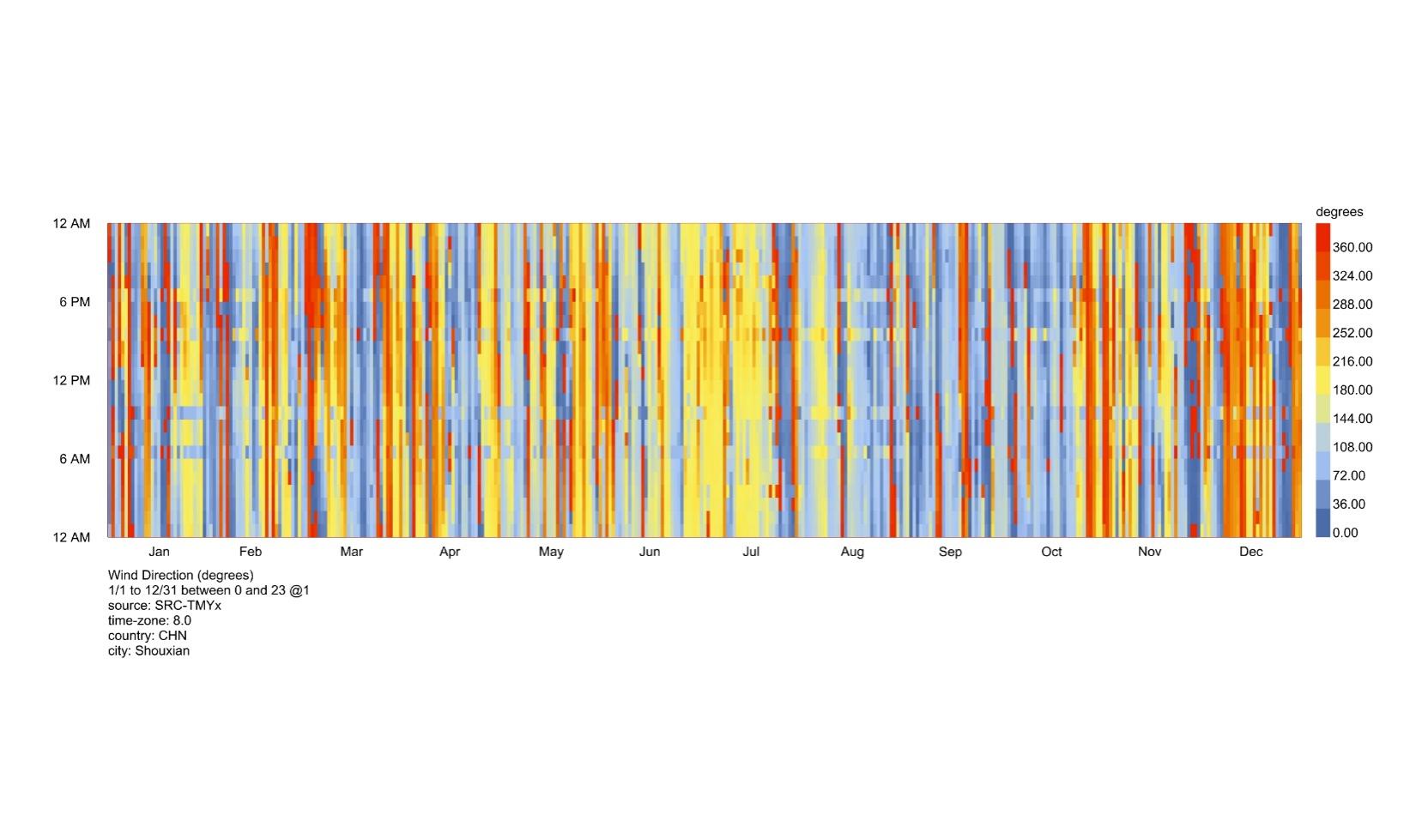


(11) Wind Direction (degrees)


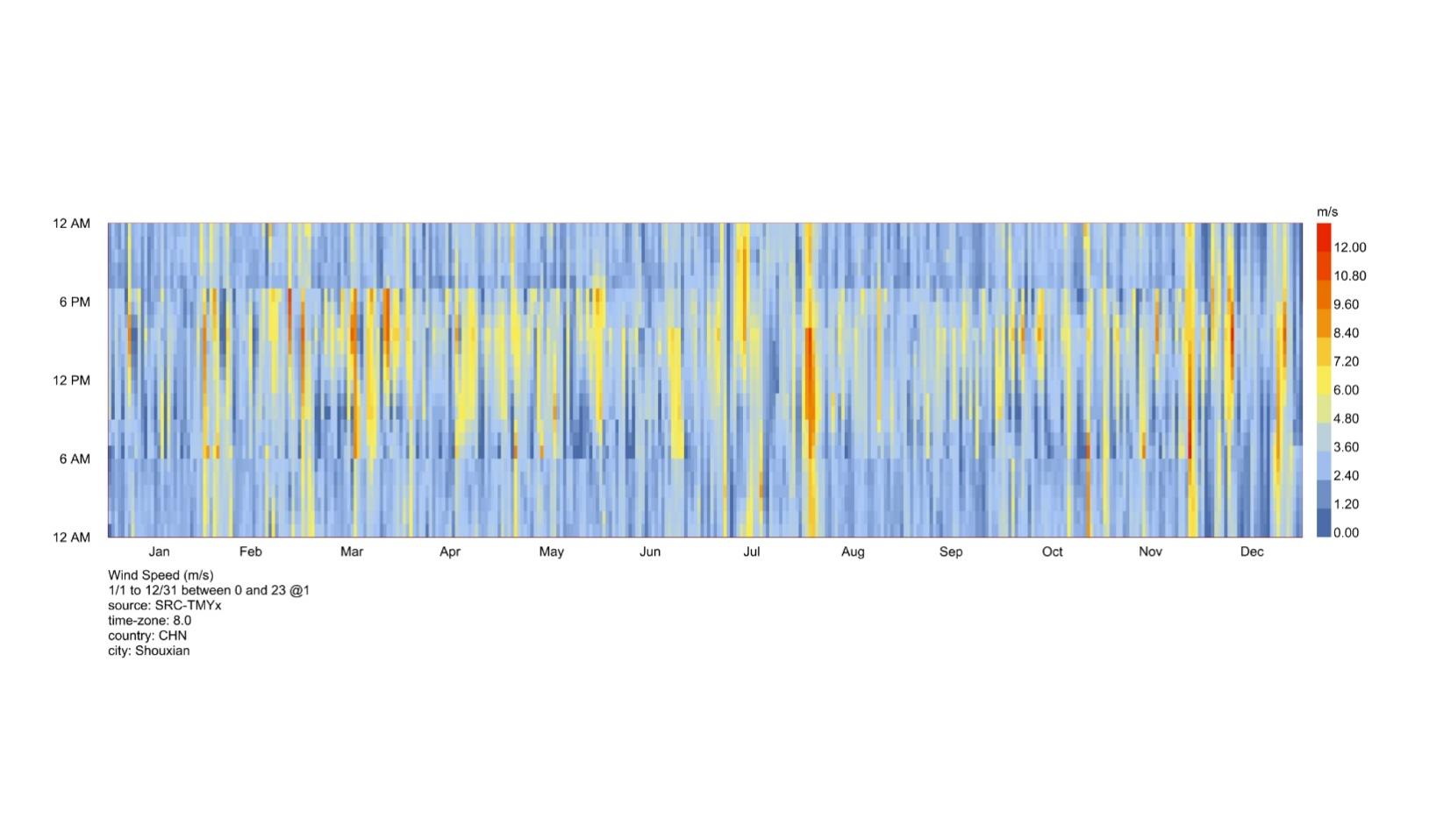


(12) Wind speed (m/s)
