# Supplementary material for: Metagenomic study of the microbiome and key geochemical potentials associated with architectural heritage sites: a case study of the Song Dynasty city wall in Shou County, China
Source: Front Microbiol. 2024 Oct 25;15:1453430. doi: 10.3389/fmicb.2024.1453430 (PMC11543536; doi:10.3389/fmicb.2024.1453430)
Supplement: Supplementary file 3 [file Data_Sheet_2.docx]

Metagenomic Study of the Microbiome and Key Geochemical Potentials Associated with Architectural Heritage Sites: A Case Study of the Song Dynasty City Wall in Shou County, China

Mingyi Zhao ^1^† , Yanyu Li ^1^† , Huanhuan Chen ^1^† , Yile Chen ^1^† , Liang Zheng ^1^ , Yue Wu ^2^ , Kang Wang ^3^ , Zhao Pan ^4,^* , Tao Yu ^5,^* , Tao Wang ^6,^*

^1^ Faculty of Humanities and Arts, Macau University of Science and Technology. Avenida Wai Long, Taipa, Macau 999078, China

^2^ Shanghai Biogenuinetech Co., Ltd. Room 304, Building 4, Lane 58 Zhanling Rd, Pudong New District, 200137, Shanghai, China

^3^ College of Life Sciences, Qingdao University. No.308 Ningxia Road, Qingdao 266000, Shandong Province, China

^4^ School of Art & Design, Shandong Jiaotong University. No.5001 Haitang Road, Changqing University Science and Technology Park, Jinan 250300, Shandong Province, China

^5^ Institutes for Translational Medicine, Qingdao University. No.308 Ningxia Road, Qingdao 266000, Shandong Province, China

^6^ The Affiliated hospital of Qingdao University, No. 16 Jiangsu Road, Qingdao 266000, Shandong Province, China

^†^ These authors have contributed equally to this work and share first authorship.

*** Correspondence:**

panzhao2024@163.com (Zhao Pan); qumea0532@163.com (Tao Yu); wangtao59@qdu.edu.cn (Tao Wang)

**Appendix B: 47 KEGG categories related to geomicrobial cycles**

The following table provides detailed information on 47 KEGG categories related to geomicrobial cycles.

**Table B1.** 47 KEGG categories related to geomicrobial cycles.

| Pathway | k_number | Detail | East-Bad-ancient-citywall_clean | East-Good-ancient-citywall_clean | North-Bad-ancient-citywall_clean | West-Good-morden-citywall_clean |
| --- | --- | --- | --- | --- | --- | --- |
| Photosystem II (psbABCDEF) | K02703 | psbA; photosystem II P680 reaction center D1 protein | 0 | 0 | 0 | 148.0194 |
| Photosystem II (psbABCDEF) | K02706 | psbD; photosystem II P680 reaction center D2 protein | 0 | 0 | 0 | 58.88326 |
| Photosystem II (psbABCDEF) | K02705 | psbC; photosystem II CP43 chlorophyll apoprotein | 0 | 0 | 0 | 126.9506 |
| Photosystem II (psbABCDEF) | K02704 | psbB; photosystem II CP47 chlorophyll apoprotein | 0 | 0 | 0 | 34.48624 |
| Photosystem II (psbABCDEF) | K02707 | psbE; photosystem II cytochrome b559 subunit alpha | 0 | 0 | 0 | 58.83059 |
| Photosystem II (psbABCDEF) | K02708 | psbF; photosystem II cytochrome b559 subunit beta | 0 | 0 | 0 | 41.37381 |
| Photosystem I (psaABCDEF) | K02689 | psaA; photosystem I P700 chlorophyll a apoprotein A1 | 0 | 0 | 0 | 18.44022 |
| Photosystem I (psaABCDEF) | K02690 | psaB; photosystem I P700 chlorophyll a apoprotein A2 | 0 | 0 | 0 | 56.95412 |
| Photosystem I (psaABCDEF) | K02691 | psaC; photosystem I subunit VII | 0 | 0 | 0 | 25.90826 |
| Photosystem I (psaABCDEF) | K02692 | psaD; photosystem I subunit II | 0 | 0 | 0 | 40.82436 |
| Photosystem I (psaABCDEF) | K02693 | psaE; photosystem I subunit IV | 0 | 0 | 0 | 23.57871 |
| Photosystem I (psaABCDEF) | K02694 | psaF; photosystem I subunit III | 0 | 0 | 0 | 26.05231 |
| Cytochrome b6/f complex (petABCDGLMN) | K02635 | petB; cytochrome b6 | 0 | 0 | 0 | 15.15323 |
| Cytochrome b6/f complex (petABCDGLMN) | K02637 | petD; cytochrome b6-f complex subunit 4 | 0 | 0 | 0 | 90.75204 |
| Cytochrome b6/f complex (petABCDGLMN) | K02634 | petA; apocytochrome f | 0 | 0 | 0 | 39.06485 |
| Cytochrome b6/f complex (petABCDGLMN) | K02636 | petC; cytochrome b6-f complex iron-sulfur subunit | 0 | 0 | 0 | 43.50445 |
| Cytochrome b6/f complex (petABCDGLMN) | K02642 | petL; cytochrome b6-f complex subunit 6 | 0 | 0 | 0 | 21.97991 |
| Cytochrome b6/f complex (petABCDGLMN) | K02643 | petM; cytochrome b6-f complex subunit 7 | 0 | 0 | 0 | 16.67279 |
| Calvin-Benson-Bassham cycle | K00855 | PRK, prkB; phosphoribulokinase [EC:2.7.1.19] | 0 | 0 | 0 | 90.77094 |
| Calvin-Benson-Bassham cycle | K01601 | rbcL; ribulose-bisphosphate carboxylase large chain [EC:4.1.1.39] | 0 | 0 | 0 | 28.17845 |
| Calvin-Benson-Bassham cycle | K01602 | rbcS; ribulose-bisphosphate carboxylase small chain [EC:4.1.1.39] | 1342.24775 | 61.87611 | 234.066 | 65.40952 |
| Calvin-Benson-Bassham cycle | K00927 | PGK, pgk; phosphoglycerate kinase [EC:2.7.2.3] | 0 | 0 | 0 | 90.85372 |
| Calvin-Benson-Bassham cycle | K00150 | gap2; glyceraldehyde-3-phosphate dehydrogenase (NAD(P)) [EC:1.2.1.59] | 0 | 0 | 0 | 24.16877 |
| Calvin-Benson-Bassham cycle | K00134 | GAPDH, gapA; glyceraldehyde 3-phosphate dehydrogenase [EC:1.2.1.12] | 278.1138312 | 0 | 0 | 106.738 |
| Calvin-Benson-Bassham cycle | K01623 | ALDO; fructose-bisphosphate aldolase, class I [EC:4.1.2.13] | 35.38622708 | 0 | 0 | 74.78631 |
| Calvin-Benson-Bassham cycle | K01624 | FBA, fbaA; fructose-bisphosphate aldolase, class II [EC:4.1.2.13] | 1.659458588 | 46.6348 | 0 | 70.34654 |
| Calvin-Benson-Bassham cycle | K03841 | FBP, fbp; fructose-1,6-bisphosphatase I [EC:3.1.3.11] | 6.150017192 | 97.21901 | 0 | 83.88398 |
| Calvin-Benson-Bassham cycle | K02446 | glpX; fructose-1,6-bisphosphatase II [EC:3.1.3.11] | 0 | 0 | 0 | 5.691406 |
| Calvin-Benson-Bassham cycle | K00615 | E2.2.1.1, tktA, tktB; transketolase [EC:2.2.1.1] | 0 | 0 | 95.6287 | 192.7498 |
| Calvin-Benson-Bassham cycle | K01807 | rpiA; ribose 5-phosphate isomerase A [EC:5.3.1.6] | 0 | 0 | 0 | 63.71037 |
| Calvin-Benson-Bassham cycle | K01808 | rpiB; ribose 5-phosphate isomerase B [EC:5.3.1.6] | 0 | 0 | 0 | 43.17378 |
| Calvin-Benson-Bassham cycle | K03737 | por, nifJ; pyruvate-ferredoxin/flavodoxin oxidoreductase [EC:1.2.7.1 1.2.7.-] | 0 | 0 | 0 | 13.69179 |
| Calvin-Benson-Bassham cycle | K01007 | pps, ppsA; pyruvate, water dikinase [EC:2.7.9.2] | 0 | 0 | 0 | 98.44547 |
| Calvin-Benson-Bassham cycle | K01006 | ppdK; pyruvate, orthophosphate dikinase [EC:2.7.9.1] | 0 | 0 | 0 | 14.15835 |
| Calvin-Benson-Bassham cycle | K01595 | ppc; phosphoenolpyruvate carboxylase [EC:4.1.1.31] | 1.771121176 | 102.7758 | 151.5899 | 162.6097 |
| Calvin-Benson-Bassham cycle | K01959 | pycA; pyruvate carboxylase subunit A [EC:6.4.1.1] | 0 | 0 | 0 | 9.08225 |
| Calvin-Benson-Bassham cycle | K01960 | pycB; pyruvate carboxylase subunit B [EC:6.4.1.1] | 0 | 0 | 0 | 9.478562 |
| Calvin-Benson-Bassham cycle | K01958 | PC, pyc; pyruvate carboxylase [EC:6.4.1.1] | 0 | 0 | 0 | 13.49805 |
| Calvin-Benson-Bassham cycle | K00024 | mdh; malate dehydrogenase [EC:1.1.1.37] | 0 | 0 | 0 | 36.48039 |
| Calvin-Benson-Bassham cycle | K01676 | E4.2.1.2A, fumA, fumB; fumarate hydratase, class I [EC:4.2.1.2] | 0 | 0 | 0 | 15.21302 |
| Calvin-Benson-Bassham cycle | K01679 | E4.2.1.2B, fumC, FH; fumarate hydratase, class II [EC:4.2.1.2] | 0 | 0 | 0 | 22.80834 |
| Calvin-Benson-Bassham cycle | K01678 | E4.2.1.2AB, fumB; fumarate hydratase subunit beta [EC:4.2.1.2] | 0 | 0 | 0 | 2.047962 |
| Calvin-Benson-Bassham cycle | K00239 | sdhA, frdA; succinate dehydrogenase / fumarate reductase, flavoprotein subunit [EC:1.3.5.1 1.3.5.4] | 0 | 0 | 0 | 66.29351 |
| Calvin-Benson-Bassham cycle | K00240 | sdhB, frdB; succinate dehydrogenase / fumarate reductase, iron-sulfur subunit [EC:1.3.5.1 1.3.5.4] | 0 | 29.4388 | 196.9182 | 180.5326 |
| Calvin-Benson-Bassham cycle | K00241 | sdhC, frdC; succinate dehydrogenase / fumarate reductase, cytochrome b subunit | 1.37474365 | 0 | 0 | 73.48678 |
| Calvin-Benson-Bassham cycle | K00242 | sdhD, frdD; succinate dehydrogenase / fumarate reductase, membrane anchor subunit | 0 | 0 | 0 | 45.91956 |
| Calvin-Benson-Bassham cycle | K01902 | sucD; succinyl-CoA synthetase alpha subunit [EC:6.2.1.5] | 0 | 0 | 0 | 77.80853 |
| Calvin-Benson-Bassham cycle | K01903 | sucC; succinyl-CoA synthetase beta subunit [EC:6.2.1.5] | 0 | 0 | 0 | 39.41011 |
| Calvin-Benson-Bassham cycle | K00174 | korA, oorA, oforA; 2-oxoglutarate/2-oxoacid ferredoxin oxidoreductase subunit alpha [EC:1.2.7.3 1.2.7.11] | 1.469104797 | 0 | 0 | 17.92953 |
| Calvin-Benson-Bassham cycle | K00175 | korB, oorB, oforB; 2-oxoglutarate/2-oxoacid ferredoxin oxidoreductase subunit beta [EC:1.2.7.3 1.2.7.11] | 1.807203786 | 0 | 0 | 48.11519 |
| Calvin-Benson-Bassham cycle | K00031 | IDH1, IDH2, icd; isocitrate dehydrogenase [EC:1.1.1.42] | 2.289367623 | 327.9436 | 182.981 | 139.3833 |
| Calvin-Benson-Bassham cycle | K01681 | ACO, acnA; aconitate hydratase [EC:4.2.1.3] | 0 | 0 | 0 | 18.77553 |
| Calvin-Benson-Bassham cycle | K01682 | acnB; aconitate hydratase 2 / 2-methylisocitrate dehydratase [EC:4.2.1.3 4.2.1.99] | 0 | 0 | 0 | 36.08199 |
| Calvin-Benson-Bassham cycle | K01491 | folD; methylenetetrahydrofolate dehydrogenase (NADP+) / methenyltetrahydrofolate cyclohydrolase [EC:1.5.1.5 3.5.4.9] | 0 | 127.711 | 360.6459 | 87.84985 |
| Calvin-Benson-Bassham cycle | K00297 | metF, MTHFR; methylenetetrahydrofolate reductase (NADPH) [EC:1.5.1.20] | 2.181840686 | 0 | 0 | 49.50675 |
| 3-hydroxypropionate bicycle | K01961 | accC; acetyl-CoA carboxylase, biotin carboxylase subunit [EC:6.4.1.2 6.3.4.14] | 0 | 0 | 0 | 50.25124 |
| 3-hydroxypropionate bicycle | K01962 | accA; acetyl-CoA carboxylase carboxyl transferase subunit alpha [EC:6.4.1.2 2.1.3.15] | 1.550793398 | 0 | 0 | 55.81465 |
| 3-hydroxypropionate bicycle | K01963 | accD; acetyl-CoA carboxylase carboxyl transferase subunit beta [EC:6.4.1.2 2.1.3.15] | 0 | 0 | 0 | 49.38596 |
| 3-hydroxypropionate bicycle | K02160 | accB, bccP; acetyl-CoA carboxylase biotin carboxyl carrier protein | 1.156578552 | 0 | 0 | 73.80784 |
| 3-hydroxypropionate bicycle | K05606 | MCEE, epi; methylmalonyl-CoA/ethylmalonyl-CoA epimerase [EC:5.1.99.1] | 1.614269956 | 25.25309 | 0 | 58.95715 |
| 3-hydroxypropionate bicycle | K01847 | MUT; methylmalonyl-CoA mutase [EC:5.4.99.2] | 0 | 0 | 0 | 6.111855 |
| 3-hydroxypropionate bicycle | K01848 | E5.4.99.2A, mcmA1; methylmalonyl-CoA mutase, N-terminal domain [EC:5.4.99.2] | 0 | 0 | 0 | 31.10925 |
| 3-hydroxypropionate bicycle | K01849 | E5.4.99.2B, mcmA2; methylmalonyl-CoA mutase, C-terminal domain [EC:5.4.99.2] | 2.761021593 | 102.828 | 0 | 54.96607 |
| 3-hydroxypropionate bicycle | K00239 | sdhA, frdA; succinate dehydrogenase / fumarate reductase, flavoprotein subunit [EC:1.3.5.1 1.3.5.4] | 0 | 0 | 0 | 66.29351 |
| 3-hydroxypropionate bicycle | K00240 | sdhB, frdB; succinate dehydrogenase / fumarate reductase, iron-sulfur subunit [EC:1.3.5.1 1.3.5.4] | 0 | 29.4388 | 196.9182 | 180.5326 |
| 3-hydroxypropionate bicycle | K00241 | sdhC, frdC; succinate dehydrogenase / fumarate reductase, cytochrome b subunit | 1.37474365 | 0 | 0 | 73.48678 |
| 3-hydroxypropionate bicycle | K01679 | E4.2.1.2B, fumC, FH; fumarate hydratase, class II [EC:4.2.1.2] | 0 | 0 | 0 | 22.80834 |
| 3-hydroxypropionate bicycle | K09709 | meh; 3-methylfumaryl-CoA hydratase [EC:4.2.1.153] | 0 | 0 | 0 | 9.799705 |
| 3-hydroxypropionate bicycle | K15019 | K15019; 3-hydroxypropionyl-coenzyme A dehydratase [EC:4.2.1.116] | 0 | 0 | 0 | 5.098062 |
| 3-hydroxypropionate bicycle | K05606 | MCEE, epi; methylmalonyl-CoA/ethylmalonyl-CoA epimerase [EC:5.1.99.1] | 1.614269956 | 25.25309 | 0 | 58.95715 |
| 3-hydroxypropionate bicycle | K01848 | E5.4.99.2A, mcmA1; methylmalonyl-CoA mutase, N-terminal domain [EC:5.4.99.2] | 0 | 0 | 0 | 31.10925 |
| 3-hydroxypropionate bicycle | K01849 | E5.4.99.2B, mcmA2; methylmalonyl-CoA mutase, C-terminal domain [EC:5.4.99.2] | 2.761021593 | 102.828 | 0 | 54.96607 |
| 3-hydroxypropionate bicycle | K14534 | abfD; 4-hydroxybutyryl-CoA dehydratase / vinylacetyl-CoA-Delta-isomerase [EC:4.2.1.120 5.3.3.3] | 0 | 0 | 0 | 2.881565 |
| 3-hydroxypropionate bicycle | K15016 | K15016; enoyl-CoA hydratase / 3-hydroxyacyl-CoA dehydrogenase [EC:4.2.1.17 1.1.1.35] | 0 | 0 | 0 | 6.0681 |
| 3-hydroxypropionate bicycle | K00626 | E2.3.1.9, atoB; acetyl-CoA C-acetyltransferase [EC:2.3.1.9] | 0 | 0 | 0 | 37.44075 |
| 3-hydroxypropionate bicycle | K01007 | pps, ppsA; pyruvate, water dikinase [EC:2.7.9.2] | 0 | 0 | 0 | 98.44547 |
| 3-hydroxypropionate bicycle | K01595 | ppc; phosphoenolpyruvate carboxylase [EC:4.1.1.31] | 1.771121176 | 102.7758 | 151.5899 | 162.6097 |
| 3-hydroxypropionate bicycle | K00024 | mdh; malate dehydrogenase [EC:1.1.1.37] | 0 | 0 | 0 | 36.48039 |
| 3-hydroxypropionate bicycle | K01676 | E4.2.1.2A, fumA, fumB; fumarate hydratase, class I [EC:4.2.1.2] | 0 | 0 | 0 | 15.21302 |
| 3-hydroxypropionate bicycle | K01678 | E4.2.1.2AB, fumB; fumarate hydratase subunit beta [EC:4.2.1.2] | 0 | 0 | 0 | 2.047962 |
| 3-hydroxypropionate bicycle | K00239 | sdhA, frdA; succinate dehydrogenase / fumarate reductase, flavoprotein subunit [EC:1.3.5.1 1.3.5.4] | 0 | 0 | 0 | 66.29351 |
| 3-hydroxypropionate bicycle | K00240 | sdhB, frdB; succinate dehydrogenase / fumarate reductase, iron-sulfur subunit [EC:1.3.5.1 1.3.5.4] | 0 | 29.4388 | 196.9182 | 180.5326 |
| 3-hydroxypropionate bicycle | K00241 | sdhC, frdC; succinate dehydrogenase / fumarate reductase, cytochrome b subunit | 1.37474365 | 0 | 0 | 73.48678 |
| 3-hydroxypropionate bicycle | K01902 | sucD; succinyl-CoA synthetase alpha subunit [EC:6.2.1.5] | 0 | 0 | 0 | 77.80853 |
| 3-hydroxypropionate bicycle | K01903 | sucC; succinyl-CoA synthetase beta subunit [EC:6.2.1.5] | 0 | 0 | 0 | 39.41011 |
| 3-hydroxypropionate bicycle | K14534 | abfD; 4-hydroxybutyryl-CoA dehydratase / vinylacetyl-CoA-Delta-isomerase [EC:4.2.1.120 5.3.3.3] | 0 | 0 | 0 | 2.881565 |
| 3-hydroxypropionate bicycle | K15016 | K15016; enoyl-CoA hydratase / 3-hydroxyacyl-CoA dehydrogenase [EC:4.2.1.17 1.1.1.35] | 0 | 0 | 0 | 6.0681 |
| 3-hydroxypropionate bicycle | K00626 | E2.3.1.9, atoB; acetyl-CoA C-acetyltransferase [EC:2.3.1.9] | 0 | 0 | 0 | 37.44075 |
| 3-hydroxypropionate bicycle | K00845 | glk; glucokinase [EC:2.7.1.2] | 299.6134135 | 0 | 0 | 29.64359 |
| 3-hydroxypropionate bicycle | K00886 | ppgK; polyphosphate glucokinase [EC:2.7.1.63] | 0 | 0 | 0 | 9.372874 |
| 3-hydroxypropionate bicycle | K01810 | GPI, pgi; glucose-6-phosphate isomerase [EC:5.3.1.9] | 1.175852964 | 42.89398 | 111.7607 | 72.04869 |
| 3-hydroxypropionate bicycle | K13810 | tal-pgi; transaldolase / glucose-6-phosphate isomerase [EC:2.2.1.2 5.3.1.9] | 0 | 0 | 0 | 7.817297 |
| 3-hydroxypropionate bicycle | K15916 | pgi-pmi; glucose/mannose-6-phosphate isomerase [EC:5.3.1.9 5.3.1.8] | 0 | 0 | 0 | 20.91833 |
| 3-hydroxypropionate bicycle | K00850 | pfkA, PFK; 6-phosphofructokinase 1 [EC:2.7.1.11] | 0 | 0 | 0 | 7.254419 |
| 3-hydroxypropionate bicycle | K16370 | pfkB; 6-phosphofructokinase 2 [EC:2.7.1.11] | 0 | 0 | 0 | 6.396099 |
| 3-hydroxypropionate bicycle | K01623 | ALDO; fructose-bisphosphate aldolase, class I [EC:4.1.2.13] | 35.38622708 | 0 | 0 | 74.78631 |
| 3-hydroxypropionate bicycle | K01624 | FBA, fbaA; fructose-bisphosphate aldolase, class II [EC:4.1.2.13] | 1.659458588 | 46.6348 | 0 | 70.34654 |
| 3-hydroxypropionate bicycle | K11645 | fbaB; fructose-bisphosphate aldolase, class I [EC:4.1.2.13] | 0 | 0 | 0 | 45.85935 |
| 3-hydroxypropionate bicycle | K16306 | K16306; fructose-bisphosphate aldolase / 2-amino-3,7-dideoxy-D-threo-hept-6-ulosonate synthase [EC:4.1.2.13 2.2.1.10] | 0 | 0 | 0 | 4.808808 |
| 3-hydroxypropionate bicycle | K01803 | TPI, tpiA; triosephosphate isomerase (TIM) [EC:5.3.1.1] | 979.4870367 | 793.7334 | 0 | 146.5253 |
| 3-hydroxypropionate bicycle | K00134 | GAPDH, gapA; glyceraldehyde 3-phosphate dehydrogenase [EC:1.2.1.12] | 278.1138312 | 0 | 0 | 106.738 |
| 3-hydroxypropionate bicycle | K00150 | gap2; glyceraldehyde-3-phosphate dehydrogenase (NAD(P)) [EC:1.2.1.59] | 0 | 0 | 0 | 24.16877 |
| 3-hydroxypropionate bicycle | K00927 | PGK, pgk; phosphoglycerate kinase [EC:2.7.2.3] | 0 | 0 | 0 | 90.85372 |
| 3-hydroxypropionate bicycle | K01834 | PGAM, gpmA; 2,3-bisphosphoglycerate-dependent phosphoglycerate mutase [EC:5.4.2.11] | 500.1206455 | 86.96455 | 0 | 57.17418 |
| 3-hydroxypropionate bicycle | K15633 | gpmI; 2,3-bisphosphoglycerate-independent phosphoglycerate mutase [EC:5.4.2.12] | 1225.07765 | 23.6749 | 294.6049 | 155.1307 |
| 3-hydroxypropionate bicycle | K15634 | gpmB; probable phosphoglycerate mutase [EC:5.4.2.12] | 0 | 0 | 0 | 17.54788 |
| 3-hydroxypropionate bicycle | K15635 | apgM; 2,3-bisphosphoglycerate-independent phosphoglycerate mutase [EC:5.4.2.12] | 0 | 0 | 0 | 23.33105 |
| 3-hydroxypropionate bicycle | K01689 | ENO, eno; enolase [EC:4.2.1.11] | 2.472891897 | 119.8165 | 359.9467 | 201.2458 |
| 3-hydroxypropionate bicycle | K00873 | PK, pyk; pyruvate kinase [EC:2.7.1.40] | 0 | 86.65689 | 128.9533 | 252.6135 |
| Entner-Doudoroff pathway, glucose-6P => glyceraldehyde-3P + pyruvate | K00036 | G6PD, zwf; glucose-6-phosphate 1-dehydrogenase [EC:1.1.1.49 1.1.1.363] | 0 | 0 | 0 | 96.40753 |
| Entner-Doudoroff pathway, glucose-6P => glyceraldehyde-4P + pyruvate | K01057 | PGLS, pgl, devB; 6-phosphogluconolactonase [EC:3.1.1.31] | 0 | 0 | 0 | 55.66206 |
| Entner-Doudoroff pathway, glucose-6P => glyceraldehyde-5P + pyruvate | K07404 | pgl; 6-phosphogluconolactonase [EC:3.1.1.31] | 0 | 0 | 0 | 27.36713 |
| Entner-Doudoroff pathway, glucose-6P => glyceraldehyde-6P + pyruvate | K01690 | edd; phosphogluconate dehydratase [EC:4.2.1.12] | 0 | 0 | 0 | 6.468632 |
| Entner-Doudoroff pathway, glucose-6P => glyceraldehyde-7P + pyruvate | K01625 | eda; 2-dehydro-3-deoxyphosphogluconate aldolase / (4S)-4-hydroxy-2-oxoglutarate aldolase [EC:4.1.2.14 4.1.3.42] | 65.70070374 | 50.03279 | 0 | 38.78577 |
| Gluconeogenesis, oxaloacetate => fructose-6P | K01596 | E4.1.1.32, pckA, PCK; phosphoenolpyruvate carboxykinase (GTP) [EC:4.1.1.32] | 0 | 106.8856 | 0 | 84.53066 |
| Gluconeogenesis, oxaloacetate => fructose-7P | K01610 | E4.1.1.49, pckA; phosphoenolpyruvate carboxykinase (ATP) [EC:4.1.1.49] | 3.787308154 | 101.6502 | 265.3601 | 118.6893 |
| Gluconeogenesis, oxaloacetate => fructose-8P | K01689 | ENO, eno; enolase [EC:4.2.1.11] | 2.472891897 | 119.8165 | 359.9467 | 201.2458 |
| Gluconeogenesis, oxaloacetate => fructose-9P | K01834 | PGAM, gpmA; 2,3-bisphosphoglycerate-dependent phosphoglycerate mutase [EC:5.4.2.11] | 500.1206455 | 86.96455 | 0 | 57.17418 |
| Gluconeogenesis, oxaloacetate => fructose-10P | K15633 | gpmI; 2,3-bisphosphoglycerate-independent phosphoglycerate mutase [EC:5.4.2.12] | 1225.07765 | 23.6749 | 294.6049 | 155.1307 |
| Gluconeogenesis, oxaloacetate => fructose-11P | K15634 | gpmB; probable phosphoglycerate mutase [EC:5.4.2.12] | 0 | 0 | 0 | 17.54788 |
| Gluconeogenesis, oxaloacetate => fructose-12P | K15635 | apgM; 2,3-bisphosphoglycerate-independent phosphoglycerate mutase [EC:5.4.2.12] | 0 | 0 | 0 | 23.33105 |
| Gluconeogenesis, oxaloacetate => fructose-13P | K00927 | PGK, pgk; phosphoglycerate kinase [EC:2.7.2.3] | 0 | 0 | 0 | 90.85372 |
| Gluconeogenesis, oxaloacetate => fructose-14P | K00134 | GAPDH, gapA; glyceraldehyde 3-phosphate dehydrogenase [EC:1.2.1.12] | 278.1138312 | 0 | 0 | 106.738 |
| Gluconeogenesis, oxaloacetate => fructose-15P | K00150 | gap2; glyceraldehyde-3-phosphate dehydrogenase (NAD(P)) [EC:1.2.1.59] | 0 | 0 | 0 | 24.16877 |
| Gluconeogenesis, oxaloacetate => fructose-16P | K01803 | TPI, tpiA; triosephosphate isomerase (TIM) [EC:5.3.1.1] | 979.4870367 | 793.7334 | 0 | 146.5253 |
| Gluconeogenesis, oxaloacetate => fructose-17P | K01623 | ALDO; fructose-bisphosphate aldolase, class I [EC:4.1.2.13] | 35.38622708 | 0 | 0 | 74.78631 |
| Gluconeogenesis, oxaloacetate => fructose-18P | K01624 | FBA, fbaA; fructose-bisphosphate aldolase, class II [EC:4.1.2.13] | 1.659458588 | 46.6348 | 0 | 70.34654 |
| Gluconeogenesis, oxaloacetate => fructose-19P | K11645 | fbaB; fructose-bisphosphate aldolase, class I [EC:4.1.2.13] | 0 | 0 | 0 | 45.85935 |
| Gluconeogenesis, oxaloacetate => fructose-20P | K03841 | FBP, fbp; fructose-1,6-bisphosphatase I [EC:3.1.3.11] | 6.150017192 | 97.21901 | 0 | 83.88398 |
| Gluconeogenesis, oxaloacetate => fructose-21P | K02446 | glpX; fructose-1,6-bisphosphatase II [EC:3.1.3.11] | 0 | 0 | 0 | 5.691406 |
| Gluconeogenesis, oxaloacetate => fructose-22P | K01622 | K01622; fructose 1,6-bisphosphate aldolase/phosphatase [EC:4.1.2.13 3.1.3.11] | 0 | 0 | 0 | 15.31507 |
| TCA cycle | K01647 | CS, gltA; citrate synthase [EC:2.3.3.1] | 0 | 0 | 0 | 63.4686 |
| TCA cycle | K01681 | ACO, acnA; aconitate hydratase [EC:4.2.1.3] | 0 | 0 | 0 | 18.77553 |
| TCA cycle | K01682 | acnB; aconitate hydratase 2 / 2-methylisocitrate dehydratase [EC:4.2.1.3 4.2.1.99] | 0 | 0 | 0 | 36.08199 |
| TCA cycle | K00031 | IDH1, IDH2, icd; isocitrate dehydrogenase [EC:1.1.1.42] | 2.289367623 | 327.9436 | 182.981 | 139.3833 |
| TCA cycle | K00030 | IDH3; isocitrate dehydrogenase (NAD+) [EC:1.1.1.41] | 0 | 0 | 0 | 33.99528 |
| TCA cycle | K00164 | OGDH, sucA; 2-oxoglutarate dehydrogenase E1 component [EC:1.2.4.2] | 0 | 0 | 0 | 6.687356 |
| TCA cycle | K00658 | DLST, sucB; 2-oxoglutarate dehydrogenase E2 component (dihydrolipoamide succinyltransferase) [EC:2.3.1.61] | 0 | 0 | 0 | 21.43886 |
| TCA cycle | K00382 | DLD, lpd, pdhD; dihydrolipoamide dehydrogenase [EC:1.8.1.4] | 0 | 0 | 0 | 92.01209 |
| TCA cycle | K00174 | korA, oorA, oforA; 2-oxoglutarate/2-oxoacid ferredoxin oxidoreductase subunit alpha [EC:1.2.7.3 1.2.7.11] | 1.469104797 | 0 | 0 | 17.92953 |
| TCA cycle | K00175 | korB, oorB, oforB; 2-oxoglutarate/2-oxoacid ferredoxin oxidoreductase subunit beta [EC:1.2.7.3 1.2.7.11] | 1.807203786 | 0 | 0 | 48.11519 |
| TCA cycle | K01902 | sucD; succinyl-CoA synthetase alpha subunit [EC:6.2.1.5] | 0 | 0 | 0 | 77.80853 |
| TCA cycle | K01903 | sucC; succinyl-CoA synthetase beta subunit [EC:6.2.1.5] | 0 | 0 | 0 | 39.41011 |
| TCA cycle | K00235 | SDHB, SDH2; succinate dehydrogenase (ubiquinone) iron-sulfur subunit [EC:1.3.5.1] | 0 | 0 | 0 | 4.65105 |
| TCA cycle | K00239 | sdhA, frdA; succinate dehydrogenase / fumarate reductase, flavoprotein subunit [EC:1.3.5.1 1.3.5.4] | 0 | 0 | 0 | 66.29351 |
| TCA cycle | K00240 | sdhB, frdB; succinate dehydrogenase / fumarate reductase, iron-sulfur subunit [EC:1.3.5.1 1.3.5.4] | 0 | 29.4388 | 196.9182 | 180.5326 |
| TCA cycle | K00241 | sdhC, frdC; succinate dehydrogenase / fumarate reductase, cytochrome b subunit | 1.37474365 | 0 | 0 | 73.48678 |
| TCA cycle | K00242 | sdhD, frdD; succinate dehydrogenase / fumarate reductase, membrane anchor subunit | 0 | 0 | 0 | 45.91956 |
| TCA cycle | K01676 | E4.2.1.2A, fumA, fumB; fumarate hydratase, class I [EC:4.2.1.2] | 0 | 0 | 0 | 15.21302 |
| TCA cycle | K01679 | E4.2.1.2B, fumC, FH; fumarate hydratase, class II [EC:4.2.1.2] | 0 | 0 | 0 | 22.80834 |
| TCA cycle | K01678 | E4.2.1.2AB, fumB; fumarate hydratase subunit beta [EC:4.2.1.2] | 0 | 0 | 0 | 2.047962 |
| TCA cycle | K00024 | mdh; malate dehydrogenase [EC:1.1.1.37] | 0 | 0 | 0 | 36.48039 |
| TCA cycle | K00116 | mqo; malate dehydrogenase (quinone) [EC:1.1.5.4] | 0 | 0 | 0 | 29.57987 |
| TCA cycle | K08264 | hdrD; heterodisulfide reductase subunit D [EC:1.8.98.1] | 2.322718426 | 0 | 0 | 2.711448 |
| Methanogenesis, acetate => methane | K00925 | ackA; acetate kinase [EC:2.7.2.1] | 0 | 61.79045 | 0 | 100.5258 |
| Methanogenesis, acetate => methane | K13788 | pta; phosphate acetyltransferase [EC:2.3.1.8] | 0 | 0 | 0 | 17.2023 |
| Methanogenesis, acetate => methane | K01895 | ACSS1_2, acs; acetyl-CoA synthetase [EC:6.2.1.1] | 0 | 0 | 0 | 60.28535 |
| Methanogenesis, acetate => methane | K00577 | mtrA; tetrahydromethanopterin S-methyltransferase subunit A [EC:2.1.1.86] | 0 | 0 | 0 | 8.662433 |
| Methanogenesis, acetate => methane | K08264 | hdrD; heterodisulfide reductase subunit D [EC:1.8.98.1] | 2.322718426 | 0 | 0 | 2.711448 |
| Methanogenesis, CO2 => methane | K00200 | fwdA, fmdA; formylmethanofuran dehydrogenase subunit A [EC:1.2.7.12] | 0 | 0 | 0 | 2.641024 |
| Methanogenesis, CO3 => methane | K00202 | fwdC, fmdC; formylmethanofuran dehydrogenase subunit C [EC:1.2.7.12] | 0 | 0 | 0 | 9.030917 |
| Methanogenesis, CO4 => methane | K11261 | fwdE, fmdE; formylmethanofuran dehydrogenase subunit E [EC:1.2.7.12] | 0 | 0 | 0 | 1.550022 |
| Methanogenesis, CO5 => methane | K00320 | mer; 5,10-methylenetetrahydromethanopterin reductase [EC:1.5.98.2] | 0 | 0 | 0 | 8.562493 |
| Methanogenesis, CO6 => methane | K00577 | mtrA; tetrahydromethanopterin S-methyltransferase subunit A [EC:2.1.1.86] | 0 | 0 | 0 | 8.662433 |
| Methanogenesis, CO7 => methane | K08264 | hdrD; heterodisulfide reductase subunit D [EC:1.8.98.1] | 2.322718426 | 0 | 0 | 2.711448 |
| Methanogenesis, CO8 => methane | K14083 | mttB; trimethylamine methyltransferase (non-essential) | 0 | 0 | 0 | 6.943244 |
| Methanogenesis, CO9 => methane | K10944 | pmoA-amoA; methane/ammonia monooxygenase subunit A [EC:1.14.18.3 1.14.99.39] | 0 | 66.59073 | 0 | 5.068651 |
| Methanogenesis, CO10 => methane | K10945 | pmoB-amoB; methane/ammonia monooxygenase subunit B | 0 | 0 | 0 | 5.580813 |
| Methanogenesis, CO11 => methane | K10946 | pmoC-amoC; methane/ammonia monooxygenase subunit C | 0 | 0 | 0 | 12.81373 |
| Mixed acid: lactate (Pyruvate to Lactate) | K00016 | LDH; L-lactate dehydrogenase | 1.477224333 | 0 | 0 | 0 |
| Mixed acid: formate to CO2 & H2 | K00122 | FDH; formate dehydrogenase [EC:1.17.1.9] | 0 | 0 | 0 | 5.401836 |
| Mixed acid: formate to CO2 & H3 | K00123 | fdoG, fdhF, fdwA; formate dehydrogenase major subunit [EC:1.17.1.9] | 0 | 0 | 0 | 7.680177 |
| Mixed acid: formate to CO2 & H4 | K00124 | fdoH, fdsB; formate dehydrogenase iron-sulfur subunit | 0 | 0 | 0 | 31.60464 |
| Mixed acid: formate to CO2 & H5 | K00126 | fdsD; formate dehydrogenase subunit delta [EC:1.17.1.9] | 0 | 0 | 0 | 5.175218 |
| Mixed acid: formate to CO2 & H6 | K00127 | fdoI, fdsG; formate dehydrogenase subunit gamma | 0 | 0 | 0 | 4.506985 |
| Mixed acid: acetate | K00156 | poxB; pyruvate dehydrogenase (quinone) [EC:1.2.5.1] | 0 | 0 | 0 | 8.415265 |
| Mixed acid: acetate | K01512 | acyP; acylphosphatase [EC:3.6.1.7] | 0 | 0 | 0 | 97.6106 |
| Mixed acid: acetate | K13788 | pta; phosphate acetyltransferase | 0 | 0 | 0 | 17.2023 |
| Mixed acid: acetate | K01512 | acyP; acylphosphatase [EC:3.6.1.7] | 0 | 0 | 0 | 97.6106 |
| Mixed acid: ethanol, acetate to acetylaldehyde | K00128 | aldehyde dehydrogenase (NAD+) | 0 | 0 | 0 | 17.20072 |
| Mixed acid: ethanol, acetate to acetylaldehyde | K00138 | aldB; aldehyde dehydrogenase | 0 | 0 | 0 | 7.945698 |
| Mixed acid: ethanol, acetate to acetylaldehyde | K13953 | alcohol dehydrogenase | 0 | 0 | 0 | 19.63561 |
| Mixed acid: ethanol, acetate to acetylaldehyde | K00121 | alcohol dehydrogenase | 0 | 0 | 0 | 45.87589 |
| Mixed acid: ethanol, acetate to acetylaldehyde | K00114 | exaA; alcohol dehydrogenase (cytochrome c) | 0 | 0 | 0 | 9.801393 |
| Mixed acid: succinate (phosphoenolpyruvate to succinate via oxaloacetate, malate & fumarate) | K01595 | ppc; phosphoenolpyruvate carboxylase [EC:4.1.1.31] | 1.771121176 | 102.7758 | 151.5899 | 162.6097 |
| Mixed acid: succinate (phosphoenolpyruvate to succinate via oxaloacetate, malate & fumarate) | K01596 | pckA; phosphoenolpyruvate carboxykinase (GTP) | 0 | 106.8856 | 0 | 84.53066 |
| Mixed acid: succinate (phosphoenolpyruvate to succinate via oxaloacetate, malate & fumarate) | K01610 | pckA; phosphoenolpyruvate carboxykinase (ATP) | 3.787308154 | 101.6502 | 265.3601 | 118.6893 |
| Mixed acid: succinate (phosphoenolpyruvate to succinate via oxaloacetate, malate & fumarate) | K00024 | mdh; malate dehydrogenase | 0 | 0 | 0 | 36.48039 |
| Mixed acid: succinate (phosphoenolpyruvate to succinate via oxaloacetate, malate & fumarate) | K00116 | mqo; malate dehydrogenase (quinone) | 0 | 0 | 0 | 29.57987 |
| Mixed acid: succinate (phosphoenolpyruvate to succinate via oxaloacetate, malate & fumarate) | K01676 | E4.2.1.2A, fumA, fumB; fumarate hydratase, class I [EC:4.2.1.2] | 0 | 0 | 0 | 15.21302 |
| Mixed acid: succinate (phosphoenolpyruvate to succinate via oxaloacetate, malate & fumarate) | K01679 | E4.2.1.2B, fumC, FH; fumarate hydratase, class II [EC:4.2.1.2] | 0 | 0 | 0 | 22.80834 |
| Mixed acid: succinate (phosphoenolpyruvate to succinate via oxaloacetate, malate & fumarate) | K01678 | E4.2.1.2AB, fumB; fumarate hydratase subunit beta [EC:4.2.1.2] | 0 | 0 | 0 | 2.047962 |
| Glyoxylate shunt | K01637 | aceA; isocitrate lyase | 0 | 0 | 0 | 32.99872 |
| Glyoxylate shunt | K01638 | aceB; malate synthase | 0 | 0 | 0 | 94.07898 |
| Anaplerotic genes | K00029 | maeB; malate dehydrogenase (oxaloacetate-decarboxylating) (NADP+) | 0 | 0 | 0 | 50.18108 |
| Anaplerotic genes | K00027 | ME2, sfcA, maeA; malate dehydrogenase (oxaloacetate-decarboxylating) [EC:1.1.1.38] | 0 | 0 | 0 | 36.15264 |
| Anaplerotic genes | K01958 | pyruvate carboxylase | 0 | 0 | 0 | 13.49805 |
| Anaplerotic genes | K01959 | pycA; pyruvate carboxylase subunit A [EC:6.4.1.1] | 0 | 0 | 0 | 9.08225 |
| Anaplerotic genes | K01960 | pycB; pyruvate carboxylase subunit B [EC:6.4.1.1] | 0 | 0 | 0 | 9.478562 |
| Anaplerotic genes | K01595 | ppc; phosphoenolpyruvate carboxylase | 1.771121176 | 102.7758 | 151.5899 | 162.6097 |
| Anaplerotic genes | K01610 | pckA; phosphoenolpyruvate carboxykinase (ATP) | 3.787308154 | 101.6502 | 265.3601 | 118.6893 |
| Anaplerotic genes | K01596 | pckA; phosphoenolpyruvate carboxykinase (GTP) | 0 | 106.8856 | 0 | 84.53066 |
| Dissimilatory nitrate reduction, nitrate -> nitrite (narGHI or napAB) | K00370 | narG, narZ, nxrA; nitrate reductase / nitrite oxidoreductase, alpha subunit [EC:1.7.5.1 1.7.99.-] | 0 | 0 | 0 | 7.140714 |
| Dissimilatory nitrate reduction, nitrate -> nitrite (narGHI or napAB) | K00371 | narH, narY, nxrB; nitrate reductase / nitrite oxidoreductase, beta subunit [EC:1.7.5.1 1.7.99.-] | 0 | 0 | 0 | 11.14614 |
| Dissimilatory nitrate reduction, nitrate -> nitrite (narGHI or napAB) | K00374 | narI, narV; nitrate reductase gamma subunit [EC:1.7.5.1 1.7.99.-] | 0 | 43.81128 | 0 | 10.19586 |
| Dissimilatory nitrate reduction, nitrate -> nitrite (narGHI or napAB) | K02568 | napB; nitrate reductase (cytochrome), electron transfer subunit | 0 | 24.51202 | 0 | 0 |
| Dissimilatory nitrate reduction, nitrite -> ammonia (nirBD or nrfAH) | K00362 | nirB; nitrite reductase (NADH) large subunit [EC:1.7.1.15] | 0 | 0 | 0 | 8.463644 |
| Dissimilatory nitrate reduction, nitrite -> ammonia (nirBD or nrfAH) | K00363 | nirD; nitrite reductase (NADH) small subunit [EC:1.7.1.15] | 0 | 0 | 0 | 11.59522 |
| Dissimilatory nitrate reduction, nitrite -> ammonia (nirBD or nrfAH) | K03385 | nrfA; nitrite reductase (cytochrome c-552) [EC:1.7.2.2] | 0 | 0 | 0 | 45.20256 |
| Dissimilatory nitrate reduction, nitrite -> ammonia (nirBD or nrfAH) | K15876 | nrfH; cytochrome c nitrite reductase small subunit | 0 | 0 | 0 | 55.69465 |
| Assimilatory nitrate reduction, nitrate -> nitrite (narB or NR or nasAB) | K00367 | narB; ferredoxin-nitrate reductase [EC:1.7.7.2] | 0 | 0 | 0 | 30.3787 |
| Assimilatory nitrate reduction, nitrate -> nitrite (narB or NR or nasAB) | K00366 | nirA; ferredoxin-nitrite reductase [EC:1.7.7.1] | 0 | 0 | 0 | 12.31099 |
| Denitrification, nitrite -> nitric oxide (nirK or nirS) | K00368 | nirK; nitrite reductase (NO-forming) [EC:1.7.2.1] | 0 | 0 | 0 | 32.17664 |
| Denitrification, nitric oxide -> nitrous oxide (norBC) | K04561 | norB; nitric oxide reductase subunit B [EC:1.7.2.5] | 0 | 0 | 0 | 40.33727 |
| Denitrification, nitrous oxide -> nitrogen (nosZ) | K00376 | nosZ; nitrous-oxide reductase [EC:1.7.2.4] | 0 | 0 | 0 | 13.27196 |
| Nitrification, ammonia -> hydroxylamine (amoABC) | K10944 | pmoA-amoA; methane/ammonia monooxygenase subunit A [EC:1.14.18.3 1.14.99.39] | 0 | 66.59073 | 0 | 5.068651 |
| Nitrification, ammonia -> hydroxylamine (amoABC) | K10945 | pmoB-amoB; methane/ammonia monooxygenase subunit B | 0 | 0 | 0 | 5.580813 |
| Nitrification, ammonia -> hydroxylamine (amoABC) | K10946 | pmoC-amoC; methane/ammonia monooxygenase subunit C | 0 | 0 | 0 | 12.81373 |
| Nitrification, nitrite -> nitrate (nxrAB) | K00370 | narG, narZ, nxrA; nitrate reductase / nitrite oxidoreductase, alpha subunit [EC:1.7.5.1 1.7.99.-] | 0 | 0 | 0 | 7.140714 |
| Nitrification, nitrite -> nitrate (nxrAB) | K00371 | narH, narY, nxrB; nitrate reductase / nitrite oxidoreductase, beta subunit [EC:1.7.5.1 1.7.99.-] | 0 | 0 | 0 | 11.14614 |
| Nitrification, nitrite -> nitrate (nxrAB) | K00958 | sat, met3; sulfate adenylyltransferase [EC:2.7.7.4] | 0 | 0 | 0 | 42.92568 |
| Nitrification, nitrite -> nitrate (nxrAB) | K00955 | cysNC; bifunctional enzyme CysN/CysC [EC:2.7.7.4 2.7.1.25] | 0 | 0 | 0 | 12.23524 |
| Nitrification, nitrite -> nitrate (nxrAB) | K00957 | cysD; sulfate adenylyltransferase subunit 2 [EC:2.7.7.4] | 0 | 144.7307 | 0 | 56.76625 |
| Nitrification, nitrite -> nitrate (nxrAB) | K00956 | cysN; sulfate adenylyltransferase subunit 1 [EC:2.7.7.4] | 0 | 0 | 0 | 12.23524 |
| Assimilatory sulfate reduction, sulfite -> sulfide (cysJI or sir) | K00380 | sulfite reductase (NADPH) flavoprotein alpha-component [EC:1.8.1.2] | 0 | 0 | 0 | 11.48085 |
| Assimilatory sulfate reduction, sulfite -> sulfide (cysJI or sir) | K00381 | sulfite reductase (NADPH) hemoprotein beta-component [EC:1.8.1.2] | 0 | 0 | 0 | 30.17602 |
| Assimilatory sulfate reduction, sulfite -> sulfide (cysJI or sir) | K00392 | sulfite reductase (ferredoxin) [EC:1.8.7.1] | 0 | 0 | 0 | 24.9285 |
| Dissimilatory sulfate reduction, sulfate -> sulfite (reversible) (sat and aprAB) | K00956 | cysN; sulfate adenylyltransferase subunit 1 [EC:2.7.7.4] | 0 | 0 | 0 | 12.23524 |
| Dissimilatory sulfate reduction, sulfate -> sulfite (reversible) (sat and aprAB) | K00957 | cysD; sulfate adenylyltransferase subunit 2 [EC:2.7.7.4] | 0 | 144.7307 | 0 | 56.76625 |
| Dissimilatory sulfate reduction, sulfate -> sulfite (reversible) (sat and aprAB) | K00958 | sat, met3; sulfate adenylyltransferase [EC:2.7.7.4] | 0 | 0 | 0 | 42.92568 |
| Dissimilatory sulfate reduction, sulfate -> sulfite (reversible) (sat and aprAB) | K00394 | aprA; adenylylsulfate reductase, subunit A [EC:1.8.99.2] | 0 | 0 | 0 | 13.18619 |
| Dissimilatory sulfate reduction, sulfate -> sulfite (reversible) (sat and aprAB) | K17226 | soxY; sulfur-oxidizing protein SoxY | 0 | 0 | 194.286 | 4.979488 |
| Dissimilatory sulfate reduction, sulfate -> sulfite (reversible) (sat and aprAB) | K16937 | doxD; thiosulfate dehydrogenase [quinone] large subunit [EC:1.8.5.2] | 0 | 0 | 0 | 33.96381 |
| Dissimilatory sulfate reduction, sulfate -> sulfite (reversible) (sat and aprAB) | K17230 | fccA; cytochrome subunit of sulfide dehydrogenase | 0 | 0 | 0 | 11.05458 |
| DMSP demethylation, MMPA -> MeSH (dmdBCD or acuH) | DmdB | MMPA-CoA ligase | 0 | 0 | 0 | 80.4452 |
| DMSP demethylation, MMPA -> MeSH (dmdBCD or acuH) | DmdC | MMPA-CoA dehydrogenase | 2.767471692 | 0 | 0 | 38.51007 |
| DMSP demethylation, MMPA -> MeSH (dmdBCD or acuH) | AcuH | acryloyl-CoA hydratase | 2.842216951 | 0 | 0 | 56.78246 |
| DMSP demethylation, MMPA -> MeSH (dmdBCD or acuH) | DddP | DMSP lyase | 0 | 0 | 0 | 17.57425 |
| MddA pathway, MeSH -> DMS (mddA) | MddA | MeSH S-methyltransferase | 0 | 0 | 0 | 3.685354 |
| MeSH oxidation, MeSH -> Formaldehyde (MTO) | MTO | MeSH oxidase | 0 | 0 | 0 | 9.671287 |
| MeSH oxidation, MeSH -> Formaldehyde (MTO) | SG_dehydratase | SG dehydratase; 6-deoxy-6-sulfo-D-gluconate dehydratase [EC:4.2.1.162] | 0 | 0 | 0 | 5.717177 |
| MeSH oxidation, MeSH -> Formaldehyde (MTO) | SLA_dehydrogenase | SLA dehydrogenase; 3-sulfolactaldehyde dehydrogenase [EC:1.2.1.97] | 0 | 0 | 0 | 27.15681 |
| MeSH oxidation, MeSH -> Formaldehyde (MTO) | SF_transaldolase | SftT; sulfofructose transaldolase; 6-deoxy-6-sulfofructose:D-glyceraldehyde-3-phosphate glyceronetransferase [EC:2.2.1.2] | 0 | 0 | 0 | 22.20324 |
| MeSH oxidation, MeSH -> Formaldehyde (MTO) | SLA_dehydrogenase | SLA dehydrogenase; 3-sulfolactaldehyde dehydrogenase [EC:1.2.1.97] | 0 | 0 | 0 | 27.15681 |
| F-type ATPase | K02111 | ATPF1A, atpA; F-type H+/Na+-transporting ATPase subunit alpha [EC:7.1.2.2 7.2.2.1] | 1.731965283 | 0 | 0 | 90.15442 |
| F-type ATPase | K02112 | ATPF1B, atpD; F-type H+/Na+-transporting ATPase subunit beta [EC:7.1.2.2 7.2.2.1] | 2.672465534 | 0 | 258.2435 | 85.31937 |
| F-type ATPase | K02113 | ATPF1D, atpH; F-type H+-transporting ATPase subunit delta | 6.087906537 | 66.72124 | 107.3226 | 107.6812 |
| F-type ATPase | K02114 | ATPF1E, atpC; F-type H+-transporting ATPase subunit epsilon | 0 | 28.04582 | 0 | 56.91534 |
| F-type ATPase | K02115 | ATPF1G, atpG; F-type H+-transporting ATPase subunit gamma | 0 | 0 | 0 | 65.66495 |
| F-type ATPase | K02108 | ATPF0A, atpB; F-type H+-transporting ATPase subunit a | 36.72856847 | 57.81804 | 0 | 103.1168 |
| F-type ATPase | K02109 | ATPF0B, atpF; F-type H+-transporting ATPase subunit b | 0 | 25.64263 | 136.5484 | 112.5237 |
| F-type ATPase | K02110 | ATPF0C, atpE; F-type H+-transporting ATPase subunit c | 0 | 0 | 0 | 169.4112 |
| F-type ATPase | K02119 | ATPVC, ntpC, atpC; V/A-type H+/Na+-transporting ATPase subunit C | 0 | 0 | 0 | 4.184612 |
| F-type ATPase | K02120 | ATPVD, ntpD, atpD; V/A-type H+/Na+-transporting ATPase subunit D | 0 | 0 | 0 | 2.299577 |
| F-type ATPase | K02122 | ATPVF, ntpF, atpF; V/A-type H+/Na+-transporting ATPase subunit F | 0 | 0 | 0 | 8.55409 |
| F-type ATPase | K02107 | ATPVG, ahaH, atpH; V/A-type H+/Na+-transporting ATPase subunit G/H | 0 | 0 | 0 | 2.951567 |
| NADH-quinone oxidoreductase | K00330 | nuoA; NADH-quinone oxidoreductase subunit A [EC:7.1.1.2] | 0 | 58.59456 | 0 | 168.3727 |
| NADH-quinone oxidoreductase | K00331 | nuoB; NADH-quinone oxidoreductase subunit B [EC:7.1.1.2] | 0 | 0 | 0 | 83.80218 |
| NADH-quinone oxidoreductase | K00332 | nuoC; NADH-quinone oxidoreductase subunit C [EC:7.1.1.2] | 0 | 0 | 0 | 26.87408 |
| NADH-quinone oxidoreductase | K00333 | nuoD; NADH-quinone oxidoreductase subunit D [EC:7.1.1.2] | 0 | 0 | 0 | 57.43647 |
| NADH-quinone oxidoreductase | K13378 | nuoCD; NADH-quinone oxidoreductase subunit C/D [EC:7.1.1.2] | 0 | 0 | 0 | 9.733008 |
| NADH-quinone oxidoreductase | K00334 | nuoE; NADH-quinone oxidoreductase subunit E [EC:7.1.1.2] | 0 | 0 | 0 | 40.47279 |
| NADH-quinone oxidoreductase | K00335 | nuoF; NADH-quinone oxidoreductase subunit F [EC:7.1.1.2] | 0 | 0 | 0 | 21.74714 |
| NADH-quinone oxidoreductase | K00337 | nuoH; NADH-quinone oxidoreductase subunit H [EC:7.1.1.2] | 0 | 0 | 0 | 92.97427 |
| NADH-quinone oxidoreductase | K00338 | nuoI; NADH-quinone oxidoreductase subunit I [EC:7.1.1.2] | 34.54797986 | 110.9251 | 0 | 165.2189 |
| NADH-quinone oxidoreductase | K00339 | nuoJ; NADH-quinone oxidoreductase subunit J [EC:7.1.1.2] | 0 | 0 | 0 | 102.0649 |
| NADH-quinone oxidoreductase | K00340 | nuoK; NADH-quinone oxidoreductase subunit K [EC:7.1.1.2] | 0.802657851 | 109.0345 | 0 | 178.4969 |
| NADH-quinone oxidoreductase | K00341 | nuoL; NADH-quinone oxidoreductase subunit L [EC:7.1.1.2] | 0 | 0 | 0 | 59.82347 |
| NADH-quinone oxidoreductase | K00342 | nuoM; NADH-quinone oxidoreductase subunit M [EC:7.1.1.2] | 0 | 0 | 0 | 64.21517 |
| NADH-quinone oxidoreductase | K00343 | nuoN; NADH-quinone oxidoreductase subunit N [EC:7.1.1.2] | 0 | 0 | 0 | 89.01089 |
| NAD(P)H-quinone oxidoreductase | K05574 | ndhC; NAD(P)H-quinone oxidoreductase subunit 3 | 0 | 0 | 0 | 16.04434 |
| NAD(P)H-quinone oxidoreductase | K05582 | ndhK; NAD(P)H-quinone oxidoreductase subunit K | 0 | 0 | 0 | 25.66364 |
| NAD(P)H-quinone oxidoreductase | K05581 | ndhJ; NAD(P)H-quinone oxidoreductase subunit J | 0 | 0 | 0 | 25.26269 |
| NAD(P)H-quinone oxidoreductase | K05579 | ndhH; NAD(P)H-quinone oxidoreductase subunit H | 0 | 0 | 0 | 39.33061 |
| NAD(P)H-quinone oxidoreductase | K05572 | ndhA; NAD(P)H-quinone oxidoreductase subunit 1 | 0 | 0 | 0 | 33.63313 |
| NAD(P)H-quinone oxidoreductase | K05580 | ndhI; NAD(P)H-quinone oxidoreductase subunit I | 0 | 0 | 0 | 44.2751 |
| NAD(P)H-quinone oxidoreductase | K05578 | ndhG; NAD(P)H-quinone oxidoreductase subunit 6 | 0 | 0 | 0 | 37.77927 |
| NAD(P)H-quinone oxidoreductase | K05576 | ndhE; NAD(P)H-quinone oxidoreductase subunit 4L | 0 | 0 | 0 | 42.72856 |
| NAD(P)H-quinone oxidoreductase | K05577 | ndhF; NAD(P)H-quinone oxidoreductase subunit 5 | 0 | 0 | 0 | 81.31321 |
| NAD(P)H-quinone oxidoreductase | K05575 | ndhD; NAD(P)H-quinone oxidoreductase subunit 4 | 0 | 0 | 0 | 85.36321 |
| NAD(P)H-quinone oxidoreductase | K05573 | ndhB; NAD(P)H-quinone oxidoreductase subunit 2 | 0 | 0 | 0 | 33.16523 |
| NAD(P)H-quinone oxidoreductase | K05583 | ndhL; NAD(P)H-quinone oxidoreductase subunit L, non-essential components | 0 | 0 | 0 | 24.24429 |
| NAD(P)H-quinone oxidoreductase | K05584 | ndhM; NAD(P)H-quinone oxidoreductase subunit M, non-essential components | 0 | 0 | 0 | 26.96906 |
| NAD(P)H-quinone oxidoreductase | K05585 | ndhN; NAD(P)H-quinone oxidoreductase subunit N, non-essential components | 0 | 0 | 0 | 33.90295 |
| Succinate dehydrogenase (ubiquinone) | K00241 | sdhC, frdC; succinate dehydrogenase / fumarate reductase, cytochrome b subunit | 1.37474365 | 0 | 0 | 73.48678 |
| Succinate dehydrogenase (ubiquinone) | K00242 | sdhD, frdD; succinate dehydrogenase / fumarate reductase, membrane anchor subunit | 0 | 0 | 0 | 45.91956 |
| Succinate dehydrogenase (ubiquinone) | K00239 | sdhA, frdA; succinate dehydrogenase / fumarate reductase, flavoprotein subunit [EC:1.3.5.1 1.3.5.4] | 0 | 0 | 0 | 66.29351 |
| Succinate dehydrogenase (ubiquinone) | K00240 | sdhB, frdB; succinate dehydrogenase / fumarate reductase, iron-sulfur subunit [EC:1.3.5.1 1.3.5.4] | 0 | 29.4388 | 196.9182 | 180.5326 |
| Cytochrome c oxidase, cbb3-type | K00404 | ccoN; cytochrome c oxidase cbb3-type subunit I | 0 | 0 | 0 | 27.83345 |
| Cytochrome c oxidase, cbb4-type | K00405 | ccoO; cytochrome c oxidase cbb3-type subunit II | 0 | 0 | 0 | 9.264321 |
| Cytochrome c oxidase, cbb5-type | K15862 | ccoNO; cytochrome c oxidase cbb3-type subunit I/II (ccoNO can replace the combination of ccoN and ccoO) | 0 | 0 | 0 | 9.560448 |
| Cytochrome c oxidase, cbb6-type | K00407 | ccoQ; cytochrome c oxidase cbb3-type subunit IV | 0 | 0 | 15.21589 | 33.1113 |
| Cytochrome c oxidase, cbb7-type | K00406 | ccoP; cytochrome c oxidase cbb3-type subunit III | 1.141743325 | 0 | 232.8932 | 29.59068 |
| Cytochrome bd ubiquinol oxidase | K00425 | cydA; cytochrome bd ubiquinol oxidase subunit I | 0 | 123.5911 | 268.5332 | 82.60901 |
| Cytochrome bd ubiquinol oxidase | K00426 | cydB; cytochrome bd ubiquinol oxidase subunit II | 0 | 213.547 | 110.0317 | 100.2804 |
| Cytochrome o ubiquinol oxidase | K02300 | cyoD; cytochrome o ubiquinol oxidase subunit IV | 0 | 0 | 0 | 14.15577 |
| Cytochrome o ubiquinol oxidase | K02299 | cyoC; cytochrome o ubiquinol oxidase subunit III | 0 | 0 | 0 | 11.28677 |
| Cytochrome o ubiquinol oxidase | K02298 | cyoB; cytochrome o ubiquinol oxidase subunit I [EC:7.1.1.3] | 0 | 0 | 0 | 13.84386 |
| Cytochrome o ubiquinol oxidase | K02297 | K02297 cyoA; cytochrome o ubiquinol oxidase subunit II [EC:7.1.1.3] | 0 | 0 | 0 | 18.96762 |
| Cytochrome c oxidase, prokaryotes | K02275 | coxB, ctaC; cytochrome c oxidase subunit II [EC:1.9.3.1] | 0 | 0 | 0 | 132.9166 |
| Cytochrome c oxidase, prokaryotes | K02274 | coxA, ctaD; cytochrome c oxidase subunit I [EC:1.9.3.1] | 0 | 0 | 0 | 58.31643 |
| Cytochrome c oxidase, prokaryotes | K02276 | coxC, ctaE; cytochrome c oxidase subunit III [EC:1.9.3.1] | 1179.982545 | 84.08287 | 276.8369 | 120.1777 |
| Cytochrome c oxidase, prokaryotes | K15408 | coxAC; cytochrome c oxidase subunit I+III [EC:1.9.3.1] | 0 | 0 | 0 | 21.09131 |
| Cytochrome c oxidase, prokaryotes | K02277 | coxD, ctaF; cytochrome c oxidase subunit IV [EC:1.9.3.1] (non-essential) | 2.999561414 | 40.1395 | 0 | 105.4838 |
| Cytochrome c oxidase, prokaryotes | K00411 | UQCRFS1, RIP1, petA; ubiquinol-cytochrome c reductase iron-sulfur subunit [EC:7.1.1.8] | 299.6027898 | 8.731729 | 19.74435 | 28.04053 |
| Cytochrome c oxidase, prokaryotes | K00412 | CYTB, petB; ubiquinol-cytochrome c reductase cytochrome b subunit | 865.5272268 | 0 | 0 | 44.49409 |
| Cytochrome c oxidase, prokaryotes | K00413 | CYC1, CYT1, petC; ubiquinol-cytochrome c reductase cytochrome c1 subunit | 3.141842999 | 1.410134 | 0 | 44.01675 |
| Cytochrome c oxidase, prokaryotes | K00416 | QCR6, UQCRH; ubiquinol-cytochrome c reductase subunit 6 | 6.483676995 | 0 | 0 | 2.134031 |
| Cytochrome c oxidase, prokaryotes | K00417 | QCR7, UQCRB; ubiquinol-cytochrome c reductase subunit 7 | 3.591187217 | 0 | 0 | 0 |
| Cytochrome c oxidase, prokaryotes | K00418 | QCR8, UQCRQ; ubiquinol-cytochrome c reductase subunit 8 | 150.9522253 | 0 | 0 | 0 |
| Cytochrome c oxidase, prokaryotes | K00420 | QCR10, UQCR; ubiquinol-cytochrome c reductase subunit 10 | 34.24319374 | 0 | 0 | 0 |
| Type I Secretion | K12340 | tolC; outer membrane protein | 0 | 0 | 0 | 85.28894 |
| Type I Secretion | K11003 | HlyD; membrane fusion protein, hemolysin D | 0 | 0 | 0 | 47.14858 |
| Type I Secretion | K11004 | HylB; ABC transpoter, ATP-binding cassette, subfamily B, bacterial HlyB | 0 | 0 | 0 | 81.31744 |
| Type I Secretion | K03226 | yscR; type III secretion protein R | 0 | 0 | 0 | 1.738421 |
| Type I Secretion | K03227 | yscS; type III secretion protein S | 0 | 0 | 0 | 1.616437 |
| Type II Secretion | K02453 | gspD; general secretion pathway protein D | 0 | 0 | 0 | 14.74024 |
| Type II Secretion | K02452 | gspC; general secretion pathway protein C | 0 | 0 | 0 | 5.210993 |
| Type II Secretion | K02455 | gspF; general secretion pathway protein F | 0 | 0 | 0 | 6.591143 |
| Type II Secretion | K02456 | gspG; general secretion pathway protein G | 0 | 0 | 0 | 96.1044 |
| Type II Secretion | K02457 | gspH; general secretion pathway protein H | 0 | 0 | 0 | 3.137013 |
| Type II Secretion | K02458 | gspI; general secretion pathway protein I | 0 | 0 | 0 | 10.32577 |
| Type II Secretion | K02459 | gspJ; general secretion pathway protein J | 0 | 0 | 0 | 8.671135 |
| Type II Secretion | K02460 | gspK; general secretion pathway protein K | 250.1402471 | 0 | 0 | 9.72123 |
| Type II Secretion | K02461 | gspL; general secretion pathway protein L | 0 | 0 | 0 | 3.722552 |
| Type II Secretion | K02462 | gspM; general secretion pathway protein M | 0 | 0 | 0 | 3.832477 |
| Type IV Secretion | K03149 | virB1; type IV secretion system protein VirB1 | 0 | 46.57479 | 0 | 96.83773 |
| Type IV Secretion | K03197 | virB2; type IV secretion system protein VirB27 | 0 | 0 | 0 | 7.546274 |
| Type IV Secretion | K03198 | virB3; type IV secretion system protein VirB3 | 0 | 0 | 0 | 2.52398 |
| Type IV Secretion | K03203 | virB8; type IV secretion system protein VirB8 | 0 | 0 | 0 | 2.603176 |
| Type IV Secretion | K03195 | virB10; type IV secretion system protein VirB10 | 452.2958967 | 0 | 0 | 0 |
| Type IV Secretion | K03205 | virD4; type IV secretion system protein VirD4 | 3.535830195 | 0 | 0 | 112.2791 |
| Type VI Secretion | K11904 | vgrG; type VI secretion system secreted protein VgrG | 0 | 85.07171 | 430.4062 | 107.082 |
| Type VI Secretion | K11903 | hcp; type VI secretion system secreted protein Hcp | 0 | 0 | 0 | 35.46052 |
| Type VI Secretion | K11906 | lip; type VI secretion system protein Lip | 0 | 0 | 0 | 22.18082 |
| Type VI Secretion | K11891 | icmF; type VI secretion system protein IcmF | 0 | 57.80365 | 0 | 37.00075 |
| Type VI Secretion | K11892 | dotU; type VI secretion system protein DotU | 0 | 11.90657 | 79.29208 | 38.89074 |
| Type VI Secretion | K11907 | clpV; type VI secretion system protein ClpV | 0 | 0 | 0 | 8.177748 |
| Sec-SRP | K03072 | secD; preprotein translocase subunit SecD | 0 | 0 | 0 | 39.78561 |
| Sec-SRP | K03074 | secF; preprotein translocase subunit SecF | 0 | 0 | 0 | 75.67039 |
| Sec-SRP | K12257 | secDF; SecD/SecF fusion protein | 0 | 0 | 0 | 15.83159 |
| Sec-SRP | K03073 | secE; preprotein translocase subunit SecE | 31.11224002 | 24.577 | 0 | 201.0507 |
| Sec-SRP | K03075 | secG; preprotein translocase subunit SecG | 1.676835912 | 30.38998 | 0 | 146.4322 |
| Sec-SRP | K03076 | secY; preprotein translocase subunit SecY | 0 | 0 | 0 | 168.7007 |
| Sec-SRP | K03210 | yajC; preprotein translocase subunit YajC | 0 | 49.60917 | 0 | 50.50182 |
| Sec-SRP | K03217 | yidC; preprotein translocase subunit YidC | 0 | 236.9504 | 176.7021 | 179.3585 |
| Sec-SRP | K03070 | secA; preprotein translocase subunit SecG | 2.028442166 | 79.82503 | 0 | 148.8867 |
| Sec-SRP | K03110 | ftsY; fused signal recognition particle receptor | 0 | 0 | 0 | 59.30231 |
| Sec-SRP | K03071 | secB; preprotein translocase subunit SecB | 0 | 105.5772 | 296.032 | 25.16194 |
| Sec-SRP | K03106 | ffh; signal recognition particle subunit Ffh | 0 | 80.41758 | 0 | 87.11109 |
| Twin arginine targeting | K03116 | tatA; sec-independent protein translocase protein TatA | 0 | 100.6386 | 211.058 | 160.0113 |
| Twin arginine targeting | K03117 | tatB; sec-independent protein translocase protein TatB | 0 | 0 | 0 | 32.78558 |
| Twin arginine targeting | K03118 | tatC; sec-independent protein translocase protein TatC | 71.36343495 | 128.8321 | 590.4765 | 179.7747 |
| Twin arginine targeting | K11017 | shlB; Type Vb, hemolysin activation/secretion protein | 0 | 0 | 0 | 9.01768 |
| Twin arginine targeting | K11016 | shlA; Type Vb, hemolysin | 0 | 0 | 0 | 4.863516 |
| Bacterial chemotaxis | K03406 | mcp; methyl-accepting chemotaxis protein | 139.7975387 | 416.6116 | 2052.208 | 471.1831 |
| Bacterial chemotaxis | K03776 | aer; aerotaxis receptor | 0 | 0 | 0 | 17.03361 |
| Bacterial chemotaxis | K10439 | rbsB; ribose transport system substrate-binding protein | 0 | 0 | 0 | 7.290193 |
| Bacterial chemotaxis | K12368 | dppA; dipeptide transport system substrate-binding protein | 0 | 0 | 0 | 31.51979 |
| Bacterial chemotaxis | K03407 | cheA; two-component system, chemotaxis family, sensor kinase CheA [EC:2.7.13.3] | 0 | 0 | 0 | 12.5589 |
| Bacterial chemotaxis | K03408 | cheW; purine-binding chemotaxis protein CheW | 0 | 63.70077 | 0 | 22.2121 |
| Bacterial chemotaxis | K03413 | cheY; two-component system, chemotaxis family, chemotaxis protein CheY | 0 | 61.40953 | 85.39337 | 112.7328 |
| Bacterial chemotaxis | K03410 | cheC; chemotaxis protein CheC | 0 | 0 | 0 | 8.570053 |
| Bacterial chemotaxis | K03414 | cheZ; chemotaxis protein CheZ | 0 | 0 | 0 | 4.958814 |
| Bacterial chemotaxis | K03409 | cheX; chemotaxis protein CheX | 0 | 0 | 0 | 3.593975 |
| Bacterial chemotaxis | K03412 | cheB; two-component system, chemotaxis family, protein-glutamate methylesterase/glutaminase [EC:3.1.1.61 3.5.1.44] | 0 | 13.66468 | 0 | 82.04184 |
| Bacterial chemotaxis | K13924 | cheBR; two-component system, chemotaxis family, CheB/CheR fusion protein [EC:2.1.1.80 3.1.1.61] | 0 | 0 | 0 | 84.26512 |
| Bacterial chemotaxis | K03415 | cheV; two-component system, chemotaxis family, chemotaxis protein CheV | 0 | 0 | 0 | 27.80372 |
| Bacterial chemotaxis | K03411 | cheD; chemotaxis protein CheD [EC:3.5.1.44] | 0 | 0 | 0 | 6.927932 |
| Bacterial chemotaxis | K00575 | cheR; chemotaxis protein methyltransferase CheR [EC:2.1.1.80] | 0 | 0 | 0 | 6.619499 |
| Bacterial chemotaxis | K02410 | fliG; flagellar motor switch protein FliG | 0 | 0 | 0 | 31.35139 |
| Bacterial chemotaxis | K02416 | fliM; flagellar motor switch protein FliM | 0 | 38.69723 | 0 | 21.11477 |
| Bacterial chemotaxis | K02417 | fliNY, fliN; flagellar motor switch protein FliN/FliY | 0 | 0 | 50.17472 | 17.11091 |
| Bacterial chemotaxis | K02556 | motA; chemotaxis protein MotA | 361.8160391 | 50.16195 | 0 | 45.81361 |
| Bacterial chemotaxis | K02557 | motB; chemotaxis protein MotB | 1.918183423 | 0 | 0 | 40.77774 |
| Bacterial chemotaxis | K02398 | flgM; negative regulator of flagellin synthesis FlgM | 0 | 51.73425 | 0 | 10.04051 |
| Bacterial chemotaxis | K02405 | fliA; RNA polymerase sigma factor for flagellar operon FliA | 0 | 113.4169 | 0 | 26.76925 |
| Bacterial chemotaxis | K02406 | fliC; flagellin | 0 | 44.85483 | 0 | 41.83675 |
| Bacterial chemotaxis | K02407 | fliD; flagellar hook-associated protein 2 | 0 | 0 | 105.9067 | 20.90801 |
| Bacterial chemotaxis | K02397 | flgL; flagellar hook-associated protein 3 FlgL | 0 | 0 | 0 | 12.37001 |
| Bacterial chemotaxis | K02396 | flgK; flagellar hook-associated protein 1 FlgK | 0 | 45.04816 | 0 | 16.37487 |
| Bacterial chemotaxis | K02414 | fliK; flagellar hook-length control protein FliK | 0 | 33.62511 | 0 | 3.373669 |
| Bacterial chemotaxis | K02389 | flgD; flagellar basal-body rod modification protein FlgD | 695.9648358 | 4.136848 | 0 | 17.20664 |
| Bacterial chemotaxis | K02390 | flgE; flagellar hook protein FlgE | 0 | 51.03976 | 0 | 23.56052 |
| Bacterial chemotaxis | K02391 | flgF; flagellar basal-body rod protein FlgF | 0 | 0 | 0 | 7.788836 |
| Bacterial chemotaxis | K02392 | flgG; flagellar basal-body rod protein FlgG | 0 | 0 | 0 | 12.29672 |
| Bacterial chemotaxis | K02393 | flgH; flagellar L-ring protein precursor FlgH | 0 | 0 | 654.331 | 13.60311 |
| Bacterial chemotaxis | K02394 | flgI; flagellar P-ring protein precursor FlgI | 0 | 61.58506 | 218.1286 | 21.28748 |
| Bacterial chemotaxis | K02387 | flgB; flagellar basal-body rod protein FlgB | 509.4816736 | 8.58531 | 0 | 9.141844 |
| Bacterial chemotaxis | K02388 | flgC; flagellar basal-body rod protein FlgC | 0 | 48.00381 | 0 | 17.77712 |
| Bacterial chemotaxis | K02408 | fliE; flagellar hook-basal body complex protein FliE | 0 | 0 | 0 | 26.85416 |
| Bacterial chemotaxis | K02409 | fliF; flagellar M-ring protein FliF | 0 | 0 | 209.5006 | 18.49526 |
| Bacterial chemotaxis | K02410 | fliG; flagellar motor switch protein FliG | 0 | 0 | 0 | 31.35139 |
| Bacterial chemotaxis | K02416 | fliM; flagellar motor switch protein FliM | 0 | 38.69723 | 0 | 21.11477 |
| Bacterial chemotaxis | K02417 | fliNY, fliN; flagellar motor switch protein FliN/FliY | 0 | 0 | 50.17472 | 17.11091 |
| Bacterial chemotaxis | K02400 | flhA; flagellar biosynthesis protein FlhA | 0 | 0 | 0 | 9.086047 |
| Bacterial chemotaxis | K02411 | fliH; flagellar assembly protein FliH | 137.2501292 | 57.14937 | 0 | 8.437503 |
| Bacterial chemotaxis | K02412 | fliI; flagellum-specific ATP synthase [EC:7.4.2.8] | 0 | 0 | 0 | 5.118542 |
| Bacterial chemotaxis | K02418 | fliOZ, fliO; flagellar protein FliO/FliZ | 0 | 0 | 0 | 4.649485 |
| Bacterial chemotaxis | K02419 | fliP; flagellar biosynthetic protein FliP | 0 | 0 | 237.2662 | 17.2099 |
| Bacterial chemotaxis | K02420 | fliQ; flagellar biosynthetic protein FliQ | 0 | 81.38045 | 0 | 19.48996 |
| Bacterial chemotaxis | K02421 | fliR; flagellar biosynthetic protein FliR | 0 | 0 | 0 | 14.39485 |
| Bacterial chemotaxis | K02556 | motA; chemotaxis protein MotA | 361.8160391 | 50.16195 | 0 | 45.81361 |
| Bacterial chemotaxis | K02557 | motB; chemotaxis protein MotB | 1.918183423 | 0 | 0 | 40.77774 |
| Bacterial chemotaxis | K02399 | flgN; flagella synthesis protein FlgN | 0 | 32.79388 | 0 | 3.144344 |
| Bacterial chemotaxis | K02413 | fliJ; flagellar FliJ protein | 0 | 22.0539 | 361.1153 | 10.39618 |
| Bacterial chemotaxis | K02422 | fliS; flagellar protein FliS | 0 | 69.16178 | 167.1031 | 11.31154 |
| Bacterial chemotaxis | K02423 | fliT; flagellar protein FliT | 0 | 28.30013 | 151.4718 | 11.64494 |
| Bacterial chemotaxis | K02386 | flgA; flagella basal body P-ring formation protein FlgA | 0 | 0 | 71.85017 | 9.629571 |
| Dissimilatory arsenic reduction (arsRBC or arsRDABC) | K00537 | arsC; arsenate reductase | 0 | 41.04529 | 0 | 42.382 |
| Dissimilatory arsenic reduction (arsRBC or arsRDABC) | K03741 | arsC; arsenate reductase | 0 | 0 | 0 | 108.6695 |
| Dissimilatory arsenic reduction (arsRBC or arsRDABC) | K03325 | arsB; arsenite transporter OR K03893 arsB; arsenical pump membrane protein | 0 | 20.9793 | 0 | 149.6975 |
| Dissimilatory arsenic reduction (arsRBC or arsRDABC) | K03893 | arsB; arsenical pump membrane protein | 0 | 0 | 0 | 19.56912 |
| Dissimilatory arsenic reduction (arsRBC or arsRDABC) | K03892 | arsR; ArsR family transcriptional regulator | 7.05697693 | 115.2343 | 181.7406 | 259.3937 |
| Dissimilatory arsenic reduction (arsRBC or arsRDABC) | K01551 | arsA; arsenite/tail-anchored protein-transporting ATPase | 0 | 53.726 | 0 | 115.8533 |

Source: Author’s statistics
